# Snakefly diversity in Early Cretaceous amber from Spain (Neuropterida, Raphidioptera)

**DOI:** 10.3897/zookeys.204.2740

**Published:** 2012-06-25

**Authors:** Ricardo Pérez-de la Fuente, Enrique Peñalver, Xavier Delclòs, Michael S. Engel

**Affiliations:** 1 Departament d’Estratigrafia, Paleontologia i Geociències Marines, Facultat de Geologia, Universitat de Barcelona, Martí i Franqués s/n, 08071 Barcelona, Spain; 2 Museo Geominero, Instituto Geológico y Minero de España, Ríos Rosas 23, 28003 Madrid, Spain; 3 Division of Entomology (Paleoentomology), Natural History Museum, and Department of Ecology & Evolutionary Biology, 1501 Crestline Drive – Suite 140, University of Kansas, Lawrence, 66049-2811 Kansas, USA

**Keywords:** Holometabola, taxonomy, paleontology, paleogeography, Mesozoic, Albian

## Abstract

The Albian amber from Spain presently harbors the greatest number and diversity of amber adult fossil snakeflies (Raphidioptera). Within Baissopteridae, *Baissoptera? cretaceoelectra* **sp. n.**, from the Peñacerrada I outcrop (Moraza, Burgos), is the first amber inclusion belonging to the family and described from western Eurasia, thus substantially expanding the paleogeographical range of the family formerly known from the Cretaceous of Brazil and eastern Asia. Within the family Mesoraphidiidae, *Necroraphidia arcuata* **gen. et sp. n.** and *Amarantoraphidia ventolina* **gen. et sp. n.** are described from the El Soplao outcrop (Rábago, Cantabria), whereas *Styporaphidia? hispanica* **sp. n.** and *Alavaraphidia imperterrita* **gen. et sp. n.** are describedfrom Peñacerrada I. In addition, three morphospecies are recognized from fragmentary remains. The following combinations are restored: *Yanoraphidia* gaoi Ren, 1995, **stat. rest.**, *Mesoraphidia durlstonensis* Jepson, Coram and Jarzembowski, 2009, **stat. rest.**, and *Mesoraphidia heteroneura* Ren, 1997, **stat. rest.** The singularity of this rich paleodiversity could be due to the paleogeographic isolation of the Iberian territory and also the prevalence of wildfires during the Cretaceous.

## Introduction

Raphidioptera (snakeflies) are regarded as one of the most primitive lineages of holometabolous insects, their fossil record dating back to the Early Jurassic (Grimaldi and Engel 2005). There is consensus that Raphidioptera forms a distinct clade together with Megaloptera and Neuroptera, the superorder Neuropterida. However, there is controversy whether Raphidioptera is sister to Megaloptera or to [Megaloptera + Neuroptera] (see Haring et al. 2011). Nowadays, the active, predatory larvae of snakeflies are long-lived, with a high number of instars and distinctive hibernating periods, living under the bark of trees and shrubs or in soil detritus; moreover, their pupation needs a period of cold to break diapause, and the pupae are exarate and active, a plesiotypic condition within Holometabola (Aspöck 2002). Adults are arboreal and also predatory, but short-lived (ibid.), exhibiting a prognathous head, a long pronotum, and a long ovipositor in females, features that give them a snake-like appeareance. The extant diversity of the order is relictual, as the Mesozoic diversity of Raphidioptera, as suggested by morphological disparity more-so-than total numbers, was greater than that observed today (e.g., Martynov 1925; Martynova 1961; Ponomarenko 1988, 1993; Oswald 1990; Ren 1997; Engel 2002; Engel et al. 2006; Perrichot and Engel 2007; Jepson and Jarzembowski 2008; Jepson et al. 2009, 2011). Moreover, while the group was once distributed throughout the world and in diverse habitats, today their range is contracted into the cold temperate regions of the Northern Hemisphere. The fossil record of Raphidioptera is comprised principally of compressions ranging from the Early Jurassic through the Miocene (Engel 2002). Snakefly inclusions in amber are far more uncommon (e.g., Carpenter 1956; Engel 1995; Aspöck and Aspöck 2004), particularly those in Cretaceous resins (Grimaldi 2000; Engel 2002; Perrichot and Engel 2007; Engel and Grimaldi 2008; Pérez-de la Fuente et al. 2010; Bechly and Wolf-Schwenninger 2011).

**Figure 1.**
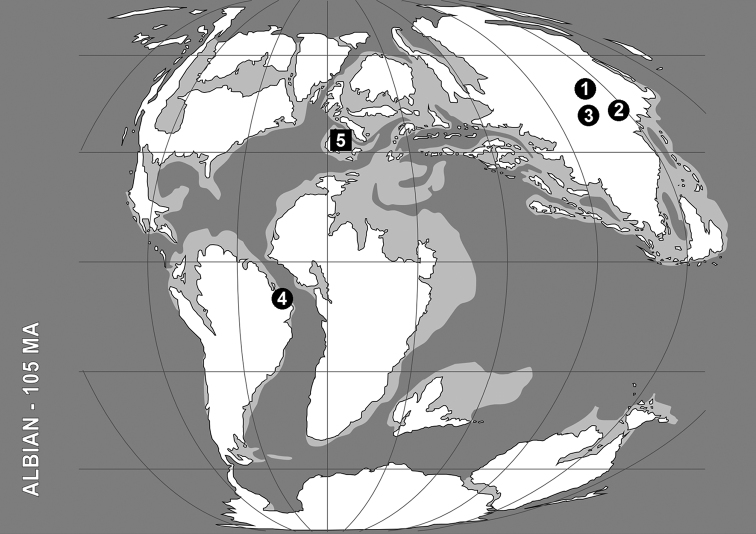
Distribution of the Early Cretaceous snakeflies currently classified within the Baissopteridae. The paleogeographic map (redrawn from Blakey 2008) corresponds to middle Albian (ca. 105 Ma). Data summarized in Engel (2002) and complemented with Jepson et al. (2011) and this paper. Circles correspond to compression localities, whereas the square represents the single amber locality in which baissopterids have been described up to date. Localities appear in chronological order after the ages summarized in Grimaldi and Engel (2005), although the age of the localities from Eastern Asia are considered generically as Early Cretaceous. Note that *Cretinocellia cellulosa* Ponomarenko, 1988 was transferred to Mesoraphidiidae s.s. by Bechly and Wolf-Schwenninger (2011) **1** Baissa, Buryat Republic, Russia (Neocomian, Valanginian). *Baissoptera cellulosa* Ponomarenko, 1993, *Baissoptera elongata* Ponomarenko, 1993, *Baissoptera kolosnitsynae* Martynova, 1961, *Baissoptera martinsoni* Martynova, 1961, *Baissoptera minima* Ponomarenko, 1993, and *Baissoptera sibirica* Ponomarenko, 1993; *Cretoraphidia certa* Ponomarenko, 1993, *Cretoraphidia macrocella* Ponomarenko, 1993, *Cretoraphidia magna* Ponomarenko, 1993, and *Cretoraphidia reticulata* Ponomarenko, 1993 **2** Liaoning, China (Neocomian: Hauterivian?). *Baissoptera euneura* Ren, 1997, *Baissoptera grandis* Ren inRen et al., 1995, and *Baissoptera liaoningensis* Ren, 1994. **3** Bon-Tsagan, Mongolia (Barremian). *Cretoraphidiopsis bontsaganensis* (Ponomarenko, 1988); *Lugala longissima* (Ponomarenko, 1988) **4** Ceará, Brazil (Aptian). *Austroraphidia brasiliensis* (Nel, Séméria, and Martins-Neto, 1990); *Baissoptera brasiliensis* Oswald, 1990, *Baissoptera pulchra* (Martins-Neto & Nel, 1992), and *Baissoptera lisae* Jepson, Ansorge and Jarzembowski, 2011 **5** Peñacerrada I (=Moraza) amber, Burgos, Spain (Albian). *Baissoptera? cretaceoelectra* sp. n.

Raphidioptera currently comprises six families, i.e., four extinct families, Mesoraphidiidae Martynov, 1925 (Late Jurassic – Late Cretaceous), Baissopteridae Martynova, 1961 (Late Jurassic – Early Cretaceous), Priscaenigmatidae Engel, 2002 (Early Jurassic; classified into its own suborder and considered the most primitive raphidiopterans), and Metaraphidiidae Bechly and Wolf-Schwenninger, 2011 (Early Jurassic); and two extant families that date back to the Eocene, Raphidiidae Latreille, 1810 and Inocelliidae Navás, 1913 (Grimaldi and Engel 2005), with ca. 210 and ca. 30 extant species respectively (Haring et al. 2011). The extinct family Baissopteridae currently comprises about 20 species distributed in five genera (Table 1). The family is considered to represent a plesiomorphic condition within Raphidioptera owing to the dense crossvenation of its representatives (Engel 2002), and it could be paraphyletic (Willmann 1994; Bechly and Wolf-Schwenninger 2011). To date, baissopterids have been exclusively recorded from Cretaceous localities, hitherto restricted to Brazil and eastern Eurasia (China, Mongolia, and Russia) (Fig. 1). The extinct family Mesoraphidiidae currently comprises about 60 species classified in 24 genera (Table 1). For an up-to-date view of the family, there is a catalog provided by Engel (2002), supplemented by Engel and Ren (2008), Jepson and Jarzembowski (2008), Jepson et al. (2009, 2011), Pérez-de la Fuente et al. (2010), and Bechly and Wolf-Schwenninger (2011). Hitherto, less than 10% of this diversity is described from amber inclusions and thus from specimens potentially more informative (Table 2). Mesoraphidiidae has been recorded from several Jurassic and Cretaceous localities primarily of the Northern Hemisphere (see Pérez-de la Fuente et al. 2010: fig. 1, supplemented by the species described from new localities by Jepson et al. 2011, Bechly and Wolf-Schwenninger 2011, and the present contribution).

**Table 1.** Currently recognized genera classified within the extinct families Baissopteridae and Mesoraphidiidae.

Family Baissopteridae Martynova, 1961
Genus *Austroraphidia* Willmann, 1994
Genus *Baissoptera* Martynova, 1961
Genus *Cretoraphidia* Ponomarenko, 1993
Genus *Cretoraphidiopsis* Engel, 2002
Genus *Lugala* Willmann, 1994
Family Mesoraphidiidae Martynov, 1925
Genus *Alavaraphidia* Pérez-de la Fuente, Peñalver, Delclòs & Engel, gen. n.
Genus *Alloraphidia* Carpenter, 1967
Genus *Amarantoraphidia* Pérez-de la Fuente, Peñalver, Delclòs & Engel, gen. n.
Genus *Archeraphidia* Ponomarenko, 1988
Genus *Baisoraphidia* Ponomarenko, 1993
Genus *Cantabroraphidia* Pérez-de la Fuente, Nel, Peñalver & Delclòs, 2010
§ Genus *Cretinocellia* Ponomarenko, 1988
Genus *Grimaldiraphidia* Bechly and Wolf-Schwenninger, 2011
Genus *Huaxiaraphidia* Hong, 1992
Genus *Iberoraphidia* Jepson, Ansorge & Jarzembowski, 2011
Genus *Jilinoraphidia* Hong and Chang, 1989
Genus *Kezuoraphidia* Willmann, 1994
Genus *Lebanoraphidia* Bechly & Wolf-Schwenninger, 2011
Genus *Mesoraphidia* Martynov, 1925
Genus *Nanoraphidia* Engel, 2002
Genus *Necroraphidia* Pérez-de la Fuente, Peñalver, Delclòs & Engel, gen. n.
Genus *Ororaphidia* Engel & Ren, 2008
Genus *Pararaphidia* Willmann, 1994
Genus *Proraphidia* Martynova, 1947
Genus *Siboptera* Ponomarenko, 1993
Genus *Sinoraphidia* Hong, 1982
Genus *Styporaphidia* Engel & Ren, 2008
Genus *Xuraphidia* Hong, 1992
Genus *Yanoraphidia* Ren, 1995

§ Recently transferred from Baissopteridae by Bechly and Wolf-Schwenninger (2011).

**Table 2.** Cretaceous amber snakeflies (Neuropterida: Raphidioptera).

Age, taxa	Amber locality (country)
Neocomian (Barremian–Aptian)	
*Lebanoraphidia nana* Bechly and Wolf-Schwenninger, 2011	Jezzine (Lebanon)
*Mesoraphidiid larva* (in Perrichot and Engel 2007)	Jezzine (Lebanon)
Albian	
*Baissoptera? cretaceoelectra* sp. n.	Peñacerrada I (Spain)
*Alavaraphidia imperterrita* gen. et sp. n.	Peñacerrada I (Spain)
*Styporaphidia? hispanica* sp. n.	Peñacerrada I (Spain)
Mesoraphidiid gen. et sp. indet. 1 (MCNA 9218)	Peñacerrada I (Spain)
Mesoraphidiid gen. et sp. indet. 3 (MCNA 9316)	Peñacerrada I (Spain)
*Cantabroraphidia marcanoi* Pérez-de la Fuente et al., 2010	El Soplao (Spain)
*Amarantoraphidia ventolina* gen. et sp. n.	El Soplao (Spain)
*Necroraphidia arcuata* gen. et sp. n.	El Soplao (Spain)
Mesoraphidiid gen. et sp. indet. 2 (CES 376)	El Soplao (Spain)
*Nanoraphidia electroburmica* Engel, 2002	Hukwang (Myanmar)
Mesoraphidiid larva 1 (in Engel 2002)	Hukwang (Myanmar)
Mesoraphidiid larva 2 (in Perrichot and Engel 2007)	Hukwang (Myanmar)
Mesoraphidiid larva 3 (in Perrichot and Engel 2007)	Hukwang (Myanmar)
Late Albian	
Mesoraphidiid larva (in Perrichot and Engel 2007)	Archingeay-Les Nouillers (France)
Turonian	
*Grimaldiraphidia luzzii* (Grimaldi, 2000)	New Jersey (USA)
Mesoraphidiid larva (in Grimaldi 2000)	New Jersey (USA)
Campanian	
Mesoraphidiid larva (in Engel and Grimaldi 2008)	Grassy Lake (Canada)

The recent discovery of a significant paleodiversity of snakeflies in Spanish amber stimulated the present work. Herein we describe this diversity and place it in the context of other Mesozoic snakeflies, highlighting some factors that could explain the uniqueness of this record when compared to other Cretaceous ambers.

## Material and methods

Samples designated by the institutional abbreviation CES are housed in the laboratory of the El Soplao Cave, Celis, Cantabria (Spain) encompassing the Institutional Collection from the El Soplao outcrop; whereas those designated as MCNA are housed in the Museo de Ciencias Naturales de Álava, Vitoria-Gasteiz, Spain. The higher classification followed herein is modified from that of Engel (2002), Perrichot and Engel (2007), and Bechly and Wolf-Schwenninger (2011). We prefer not to recognize subfamilies within Mesoraphidiidae as it is unclear what the monophyletic lineages of this complex truly comprise. Morphological terminology generally follows that of Aspöck et al. (1991). Vein and cell nomenclature is after Engel (2011): the term “radial” is used for cells bounded between R and Rs, “discal” is used for the cell bounded between Rs and MA, “medial” is used for cells bounded between MA and MP, and “discoidal” is reserved for cells bounded anteriorly by MP and posteriorly by MP or CuA (Fig. 9). Also, the number of Rs branches is considered as those that reach the wing margin. Genitalia nomenclature follows that of Aspöck and Aspöck (2008), i.e., the numbers 9, 10 or 11 accompanying abdominal/genitalic structures indicate to which abdominal segment they allegedly belong; the parameres are considered to be the fused gonocoxites, gonapophyses and gonostyli from the 10th abdominal segment; and the term trichobothria refers to the bristles located in the center of a depressed ring in the cuticle of tergite 10 (+11?) in Neuropterida.

Drawings were prepared with a camera lucida attached to an Olympus BX51 microscope. Photomicrographs were prepared using a Nikon D1x digital camera attached to an Infinity K-2 long-distance microscopic lens. Images were merged using Combine ZP and Helicon Focus 4.2.1 (HeliconSoft Ltd.) softwares. All measurements are in millimeters and were taken using an ocular graticule.

Abbreviations. Veins: A, anal; C, costa; CuA, cubital anterior; CuP, cubital posterior; MA, medial anterior; MP, medial posterior; ptc, pterostigmal crossvein; R, radial; Rs, radial sector; Sc, subcosta. Wing fields (in italics): *dcal*, discal cell; *doi*, discoidal cell; *m*, medial cell; *pt*, pterostigma; *r*, radial cell; *sr*, subradial cell.

### Geological setting

The Peñacerrada I (in Moraza, Burgos. 42°40'22"N, 2°42'57"W) and El Soplao (in Rábago, Cantabria. 43°18'20"N, 4°26'50"W) amber-bearing deposits are included within the Basque-Cantabrian Basin (BCB) in the north of the Iberian Peninsula. To the south, the BCB constitutes a thrust sheet on the Cenozoic Duero and Ebro basins, while to the north it extends offshore to the Gulf of Biscay. BCB’s oriental limit is in the Pyrenees, and its occidental limit is in the Asturian Paleozoic Massif. The evolution of the BCB during the Late Jurassic-Early Cretaceous was contextualized into a stretching rift setting related with the opening of both the North Atlantic Ocean and the Biscayan Gulf. This regional extension produced a complex distribution of sub-basins and depositional areas, with different sedimentation features between them.

The amber localities are Albian in age, about 110 Mya (Peñalver and Delclòs 2010), and are always associated with coal layers. The amber is included within the Escucha Formation (Peñacerrada I, in the oriental part of the BCB) (Martínez-Torres et al. 2003; Delclòs et al. 2007) and within Las Peñosas Formation (El Soplao, in the occidental part of the BCB) (Najarro et al. 2009, 2010). In Peñacerrada I, the amber is embedded into a siliciclastic unit with organic-rich silts, clays, and coal levels. These levels were deposited in non-marine environments, into interdistributary bays within a deltaic plain, and sometimes were related with channel infillings which drained the surrounding forests. The discontinuous presence of dinoflagellate cysts (Núñez-Betelu 1998) and orbitolinids (Martínez-Torres et al. 2003) suggests a periodical seawater influence. In El Soplao, the amber also occurs in organic-rich silt-claystones which were deposited into interdistributary and coastal bays, or marshes. This deposit is characterized by the presence of some decimetric levels of cuticle-plant remains indicating strong continental influence. They are interbedded with other levels containing brackish or marine gastropods, bivalves, bryozoans, and serpulids (Najarro et al. 2010), some of them fixed on the surfaces of the amber pieces, and this circumstance suggests a more marine-influenced environment than in the Peñacerrada I outcrop. The Albian amber occurrences in the BCB are coinciding with transitions from maximum regional regressions to deltaic progradations (Martínez-Torres et al. 2003; Najarro et al. 2009).

### Paleoecological setting

From Peñacerrada I two different vegetational assemblages were distinguished in palynological analyses (Barrón et al. 2001; Diéguez et al. 2010). One assemblage developed on the alluvial plains and was composed of a mixed conifer forest (chiefly Araucariaceae and Taxodiaceae–Cupressaceae, but also with some Pinaceae, Podocarpaceae and Ginkgoaceae) with an understory characterized by lycopsids and Schizaeaceae. The other assemblage was composed of xeromorphic vegetation that grew on coastal environments, containing Cheirolepidiaceae, Cycadophyta, Gnetophyta, and some arboreal ferns adapted to dry conditions, such as Matoniaceae and Cyatheaceae/Dicksoniaceae/Dipteridaceae. In El Soplao, the area was covered by a mixed forest that grew close to the seaside and was composed by Cheirolepidiaceae, Cupressaceae, and Miroviaceae, with an understory containing pteridophytes, cycads, ginkgoales, and/or bennettitales; swamp and pond areas were occupied by cryptogams and early angiosperms (Najarro et al. 2009, 2010).

Different tree groups have been interpreted as the resin producers. For the oriental area of the BCB (Peñacerrada I), araucariaceans close to the Recent genus *Agathis* were suggested as original producers of the resin (Alonso et al. 2000; Chaler and Grimalt 2005), while in the occidental area (El Soplao), the conifer genus *Frenelopsis*, of the extinct family Cheirolepidiaceae,plus another unidentified plant, were indicated (Menor-Salván et al. 2010; Najarro et al. 2010). The presence in both outcrops of abundant charcoal in the amber-bearing sediments and charcoalified plant fibers embedded into the amber indicates the occurrence of wildfires in the paleoenvironment (Najarro et al. 2010).

## Systematic paleontology

### Order Raphidioptera Navás, 1916. Suborder Raphidiomorpha Engel, 2002. Family Baissopteridae Martynova, 1961. Genus *Baissoptera* Martynova, 1961

#### Baissoptera ? cretaceoelectra

Pérez-de la Fuente, Peñalver, Delclòs & Engel sp. n.

urn:lsid:zoobank.org:act:94EB4ADD-CCC8-46CB-98AF-F2EC4D47BDDC

Figs 2 , 3

##### Holotype.

MCNA 12068.4, from Peñacerrada I amber; fore- and hind wing distal fragments. Three associated hymenopterans are preserved as syninclusions.

##### Diagnosis.

Fore- and hind wing with a relatively long pterostigma with a strongly oblique and slightly sinusoid pterostigmal crossvein placed beyond pterostigmal midlength; fore- and hind wing with one closed radial cell distal to pterostigma; forewing Rs with six branches; forewing with at least eight closed subradial cells.

##### Description.

Sex unknown. Veins with some strong, very short setae preserved, membrane hyaline. *Forewing*.Length of preserved fragment 6.4,maximum width 2.8;wing apex relatively rounded; pterostigma relatively long (2.4 long, length ca. eight times basal pterostigmal width), slightly widening distally, not conspicuously infumate as preserved; pterostigma with a strongly oblique and slightly sinusoid pterostigmal crossvein placed beyond pterostigmal midlength, basally closed by a crossvein; pterostigma longer than any radial cell; R with two branches beyond pterostigma;at least three radial cells present, one closed radial cell partly distal to pterostigma; Rs with six branches and at least eight closed subradial cells;MA at least with two branches; gradate seriesveryregular, almost following a staircase-like pattern. *Hind wing*.Length of preserved fragment 5.9,maximum width as preserved 2.7;wing apex more pointed than in forewing;costal field distinctly narrower than in forewing; one c-sc crossvein preserved;pterostigma relatively long (2.5 long, length ca. 10 times basal pterostigmal width), slightly widening distally, not conspicuously infumate as preserved, starting 0.5 (twice pterostigmal basal width) beyond termination of Sc; pterostigma with a strongly oblique and slightly sinusoid pterostigmal crossvein placed beyond pterostigmal midlength, basally closed by a crossvein;pterostigma longer than any radial cell;R with two branches beyond pterostigma;at least five radial cells present, one small closed radial cell distal to pterostigma;Rs with five branches and at least seven closed subradial cells; MA at least with two branches; gradate seriesveryregular, almost following a staircase-like pattern.

**Figure 2.**
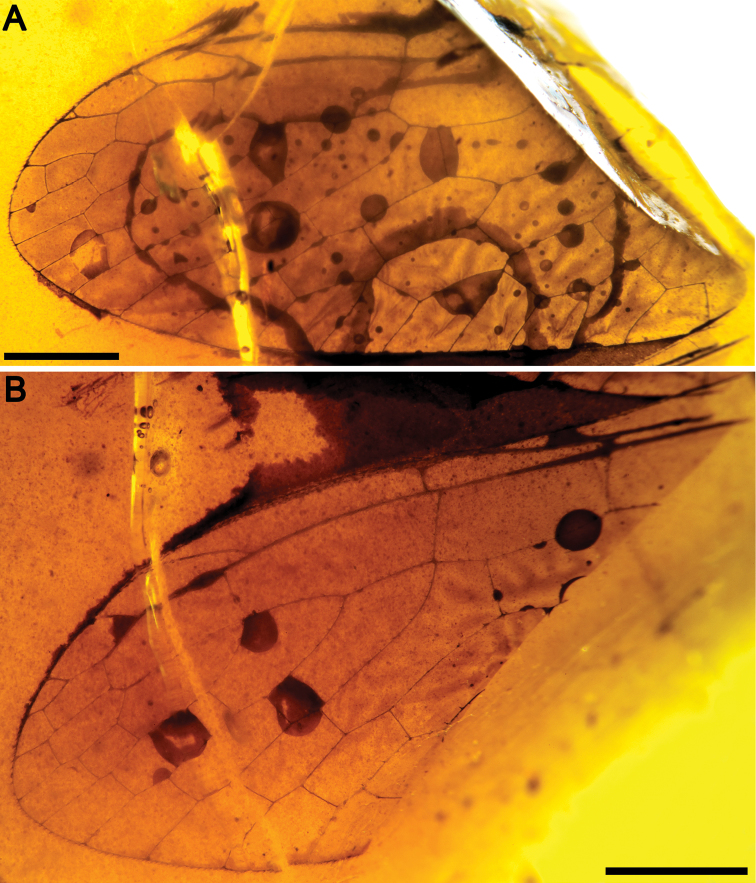
*Baissoptera? cretaceoelectra* sp. n., holotype MCNA 12068.4. **A** forewing **B** hind wing. Scale bars = 1 mm.

**Figure 3.**
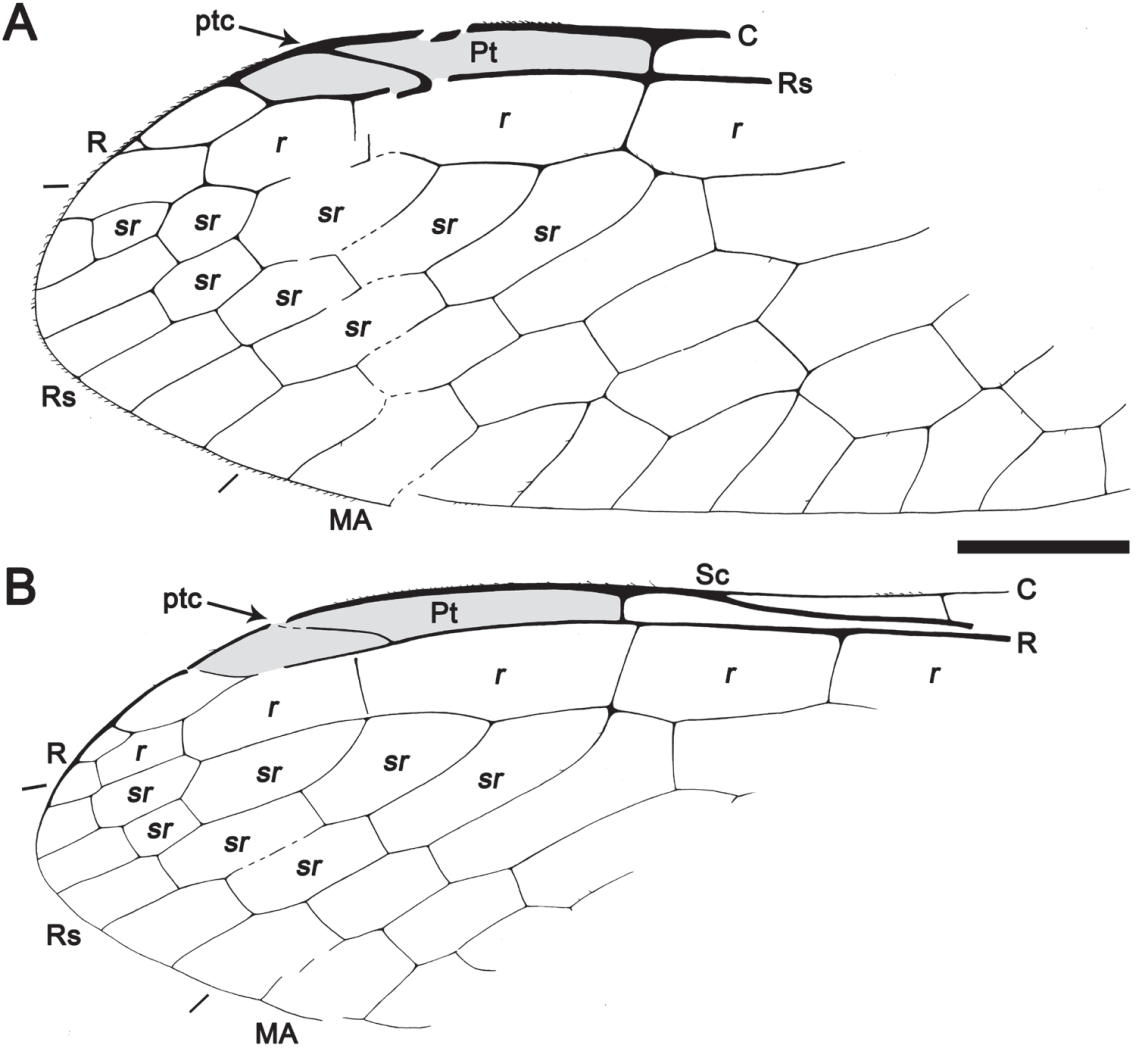
Drawings of *Baissoptera? cretaceoelectra* sp. n., holotype MCNA 12068.4. **A**, forewing **B** hind wing. Scale bar = 1 mm (both wings at the same scale).

##### Etymology.

The specific epithet is a combination of the Greek words *cretaceus* (taken from the period name, although specifically meaning “chalky”) and *elektron*, meaning “amber”.

##### Comments.

Within the current taxonomic framework, the numerous crossveins of MCNA 12068.4 are indicative of placement in the Baissopteridae. Unfortunately, neither base of the wing is preserved, thus important characters such as the maximum width of the costal field, the pattern of distribution of c-sc crossveins (= costal crossveins), the separation between M and CuA in the forewing, and the shape of the basal piece of MA, are unknown. Also, the infumation of the pterostigma is not evident in the holotype, but it is uncertain if this could have been caused by taphonomical processes and is, therefore, not used as a diagnostic character although if the absence of infumation is true of the species in life, then it would represent a remarkable difference from all other described baissopterids. Fortunately, the pterostigma can be delimited thanks to the relative parallelness of C and R (R tends to conspicuously change its slope beyond the pterostigma in the other baissopterids) and also the greater thickness of both veins.

The specimen is tentatively classified within the genus *Baissoptera* as it has the pterostigmal crossvein most similar to the diversity found within this genus. Today just two of the 12 species currently classified within the genus *Baissoptera*, i.e., *Baissoptera brasiliensis* Oswald, 1990 and *Baissoptera lisae* Jepson, Ansorge & Jarzembowski, 2011, lack a pterostigmal crossvein in both fore- and hind wings (Oswald 1990: p. 156, figs 3, 4; Jepson et al. 2011: p. 393, text-figs 6A, B). Some genera can even show an additional, straight pterostigmal crossvein in a more basal position at least in the hind wing, i.e., *Baissoptera kolosnitsynae* Martynova, 1961 and *Baissoptera pulchra* (Martins-Neto and Nel, 1992) (Martynova 1961: p. 81, fig. 7; Martins-Neto and Nel 1992: p. 428, figs 2, 3). Regarding the other taxa currently classified within the Baissopteridae, the genera *Lugala* and *Cretoraphidia* lack a pterostigmal crossvein, at least in the hind wing (Ponomarenko 1988: p. 75, fig. 4; 1993: p. 70, figs 7, 9, 10); whereas the genera *Cretoraphidiopsis* and *Austroraphidia*, althoughshowing a pterostigmal crossvein situated beyond pterostigmal midlength, have it not as strongly oblique as in *Baissoptera? cretaceoelectra* sp. n., both showing irregular gradate series in both wings and a much lesser number of Rs branches in the forewing (three in *Austroraphidia*, four in *Cretoraphidiopsis*). *Cretinocellia cellulosa* Ponomarenko, 1988 has been recently transferred from the Baissopteridae to the Mesoraphidiidae by Bechly and Wolf-Schwenninger (2011) based on its lack of pterostigmal crossvein(s) and a Sc ending about midwing length. Although these two characters are also present in *Cretoraphidia certa* Ponomarenko, 1993 and *Cretoraphidia magna* Ponomarenko, 1993 (Ponomarenko 1993: p. 70, figs 7, 10), in both, the crossvenation is relatively higher than in *Cretoraphidia cellulosa* and should therefore remain in Baissopteridae for the moment. Consequently, Bechly and Wolf-Schwenninger (2011) also noted that the genus *Cretinocellia* might occupy a basalmost position within Mesoraphidiidae according to its relatively high crossvenation compared to the other mesoraphidiids. On the other hand, we still consider *Arariperaphidia rochai* Martins-Neto and Vulcano, 1989 as incertae sedis rather than as a baissopterid (*contra* Bechly and Wolf-Schwenninger 2011), owing to its lack of preserved characters indicating a more conclusive assignment. The shape and location of the pterostigmal crossvein is quite diagnostic for *Baissoptera? cretaceoelectra*. Only *Baissoptera minima* Ponomarenko, 1993 shows such a strongly oblique pterostigmal crossvein within the family, even slightly sinusoid as in the new species, in a relatively elongate pterostigma (length ca. eight times basal pterostigmal width) (Ponomarenko 1993: p. 64, fig. 2). However, the pterostigmal crossvein is located before pterostigmal midlength and Rs is poorly branched in *Baissoptera minima*. *Baissoptera? cretaceoelectra* has Rs in the forewing with more branches currently described within the genus. The remaining *Baissoptera* species always show a lesser number of branches of Rs in the forewing, i.e., five (*Baissoptera brasiliensis*, *Baissoptera grandis* Ren in Ren et al. 1995, *Baissoptera liaoningensis* Ren, 1994, *Baissoptera lisae*, and *Baissoptera sibirica* Ponomarenko, 1993), four (*Baissoptera elongata* Ponomarenko, 1993, *Baissoptera euneura* Ren, 1997, *Baissoptera kolosnitsynae*, and *Baissoptera martinsoni* Martynova, 1961), or three Rs branches (*Baissoptera pulchra* and *Baissoptera minima*) (Martynova 1961; Oswald 1990; Martins-Neto and Nel 1992; Ponomarenko 1993; Ren 1994, 1997; Ren et al. 1995; Jepson et al. 2011). Although *Baissoptera cellulosa* Ponomarenko, 1993 (based on a forewing lacking the apex) could also possess six branches of Rs and does have a sinuate pterostigmal crossvein presumably beyond pterostigmal midlength (Ponomarenko 1993: p. 65, fig. 3), it differs from *Baissoptera? cretaceoelectra* in that the pterostigmal crossvein is just slightly oblique and the more abundant crossvenation. The only other described baissopterid with such an elevated number of branches of Rs is *Cretoraphidia certa*, but it lacks a pterostigmal crossvein as has been discussed, and in addition Sc ends in a more basal position. Furthermore, the pterostigmal length of the new species is elongate when compared to the other lengths shown by the other species within the genus *Baissoptera*. Only *Baissoptera grandis* has a longer pterostigma, its length about 11 times its basal pterostigmal width (Ren et al. 1995: p. 175, fig. 2). The shortest pterostigmata within the genus are found in *Baissoptera martinsoni* and *Baissoptera elongata*, their lengths ca. four and six times their basal pterostigmal widths, respectively (Martynova 1961: p. 80, fig. 6; Ponomarenko 1993: p. 67, fig. 5). Naturally, our placement of this species in *Baissoptera* is a conservative position based on the lack of complete material. More completely-preserved specimens, in which the wing base characters noted above could be assessed, may force a reconsideration of the generic assignment.

### Family Mesoraphidiidae Martynov, 1925

#### Necroraphidia

Pérez-de la Fuente, Peñalver, Delclòs & Engel gen. n.

urn:lsid:zoobank.org:act:BF4F7D74-232F-4F2E-8754-E3940CD9D8B0

http://species-id.net/wiki/Necroraphidia

##### Type species.

*Necroraphidia arcuata* sp. n.

##### Diagnosis.

Small size; costal field very broad; pterostigma with a single, subdistal, strongly oblique, slightly sinuose to arcuate crossvein; pterostigma with a diffuse base; forewing with Rs and MA each forked twice; forewing with second radial cell proximally broad.

##### Etymology.

The new genus-group name is a combination of the Greek word *nekros*, meaning, “dead”, and *Raphidia*, common generic stem for snakeflies. The name is feminine.

##### Comments.

**Figure 4.**
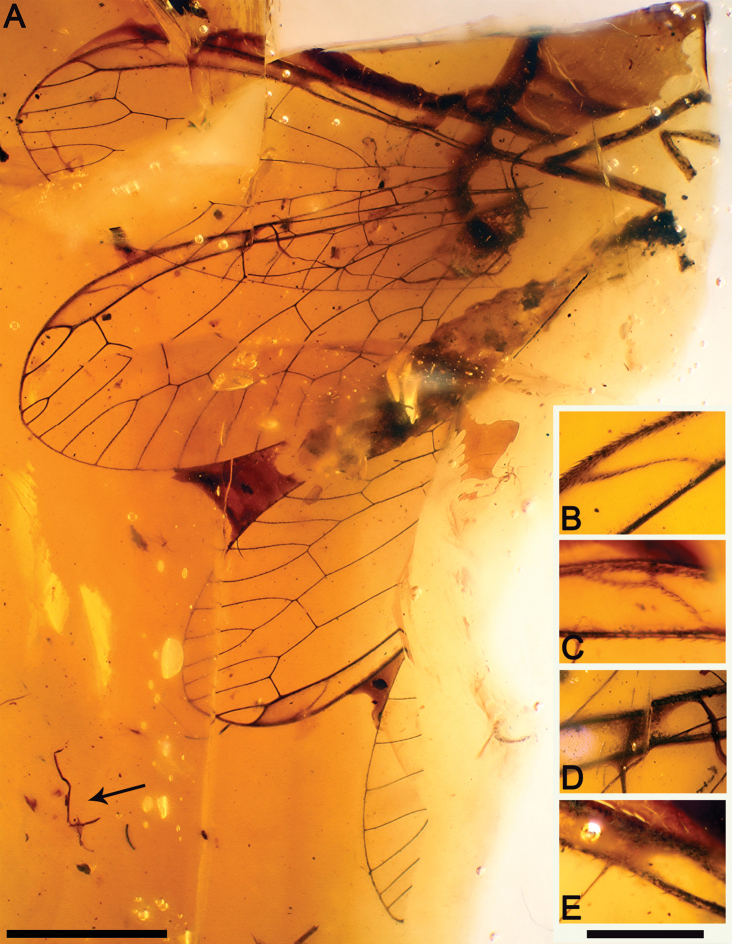
*Necroraphidia arcuata* gen. et sp. n., holotype CES 391.1. **A** ventral habitus, note some charcoalified plant fibersnearby the specimen (arrow) **B** right forewing pterostigmal crossvein **C** right hind wing pterostigmal crossvein **D** right forewing pterostigmal diffuse base **E** right hind wing pterostigmal diffuse base. Scale bars: **A** = 2 mm; **B, C, D, E** = 0.5 mm.

*Necroraphidia* gen. n. is most similar to *Ororaphidia* and *Styporaphidia* from the Late Jurassic of Inner Mongolia, China (Engel and Ren 2008). These three genera show the very diagnostic character of a diffuse base to the pterostigma, lacking a crossvein as the proximal boundary of this wing region (figs 4D–E). Also, they share the presence of, at least, one pterostigmal crossvein subdistally, three discoidal cells posterior to MP in the forewing (forming a triangle), and a larger size if compared with other minute mesoraphidiids. *Caloraphidia glossophylla* Ren, 1997 (combination restored by Bechly and Wolf-Schwenninger 2011) shares these three characters, but it lacks the diffuse pterostigmal base according to Ren (1997: p. 184, fig. 11). Hence, to consider this species as closely related (i.e., as an ororaphidiine in their system) as suggested by Bechly and Wolf-Schwenninger (2011) seems dubious. *Necroraphidia* can be separated from *Styporaphidia* by the presence of a single pterostigmal crossvein (two in *Styporaphidia*) and the three branches of both Rs and MA (two branches in *Styporaphidia*), while it can be distinguished most readily from *Ororaphidia* by the strongly oblique and arcuate shape of the pterostigmal crossvein (less oblique and straight in *Ororaphidia*), the proximally broader second radial cell (narrowly triangular proximad in *Ororaphidia*), the smaller second discoidal cell, and also by the smaller size (forewing length 11.4 in *Ororaphidia*) (Engel and Ren 2008).

#### Necroraphidia arcuata

Pérez-de la Fuente, Peñalver, Delclòs & Engel sp. n.

urn:lsid:zoobank.org:act:A0CA422D-FD60-4753-B8DE-7C5A4E75BDAE

http://species-id.net/wiki/Necroraphidia_arcuata

Figs 4 , 5

##### Holotype.

CES 391.1, from El Soplao amber; incomplete specimen, almost complete left fore- and hind wings (lacking their basalmost part), distal half of right hind wing and apex of right forewing, partial abdomen, and two leg fragments. Dense fungal hyphae infestate the abdomen and wings. The specimen is preserved together with the following syninclusions: two coleopterans, two hymenopterans (one of them, CES 391.2, belonging to the Megalyridae; Pérez-de la Fuente et al. 2012), one immature aphid, a cluster of trichomes, a fewcharcoalified plant fibers (Fig. 7A), a few timber debris, as well as other indeterminate organic remains.

##### Diagnosis.

As for the genus (*vide supra*).

##### Description.

Sex unknown.Legs patterned as follows (at least in the preserved fragments): femur with three dark areas, tibia with proximal area darkened and a dark area beyond midlength.Wing veins brown; veins with strong, very short setae, especially abundant on C; membrane hyaline. *Forewing*.Length of preserved fragment 6.9 (estimated total wing length > 9), maximum width 2.7; costal field very broad (costal field about two times wider than pterostigmal base at widest preserved point; Sc terminating into C around two-thirds of estimated wing length; three c-sc crossveins preserved; single, proximal sc-r crossvein; pterostigma elongate (3.2 long, longer than either radial cell), widening distally (maximum width almost twice basal width), and faintly infumate, starting at termination of Sc; pterostigma with a single, subdistal, strongly oblique, slightly sinuose crossvein; pterostigma with a diffuse base (i.e., lacking a crossvein as proximal boundary of this wing region); Rs with three branches, distalmost fork very short; two large radial cells present; first radial cell about 1.3 times longer than second radial cell; second radial cell proximally broad; MA arising slightly distad midpoint of first radial cell, with three branches; three discoidal cells posterior to MP; 1cua-cup crossvein not preserved; anal veins not preserved; jugal lobe not visible. *Hind wing*.Length of preserved fragment 6.6 (estimated total wing length 8–9), maximum width 2.7;costal field distinctly narrower than in forewing;four c-sc crossveins preserved;pterostigma elongate (2.9 long, longer than either radial cell), widening distally (maximum width almost twice basal width), and faintly infumate, starting at termination of Sc; pterostigma with a single, subdistal, strongly oblique, arcuate crossvein; pterostigma with a diffuse base; Rs with two branches; two radial cells present; MA with three branches; two discoidal cells posterior to MP, the first one trianguloid, not much smaller than second one; 1ma-mp crossvein not especially close to fork between Rs and MA; anal area not preserved. *Abdomen*. Length 3.8. Genitalia degraded, with dorsal part missing, and badly seen due to presence of dense fungal hyphae.

**Figure 5.**
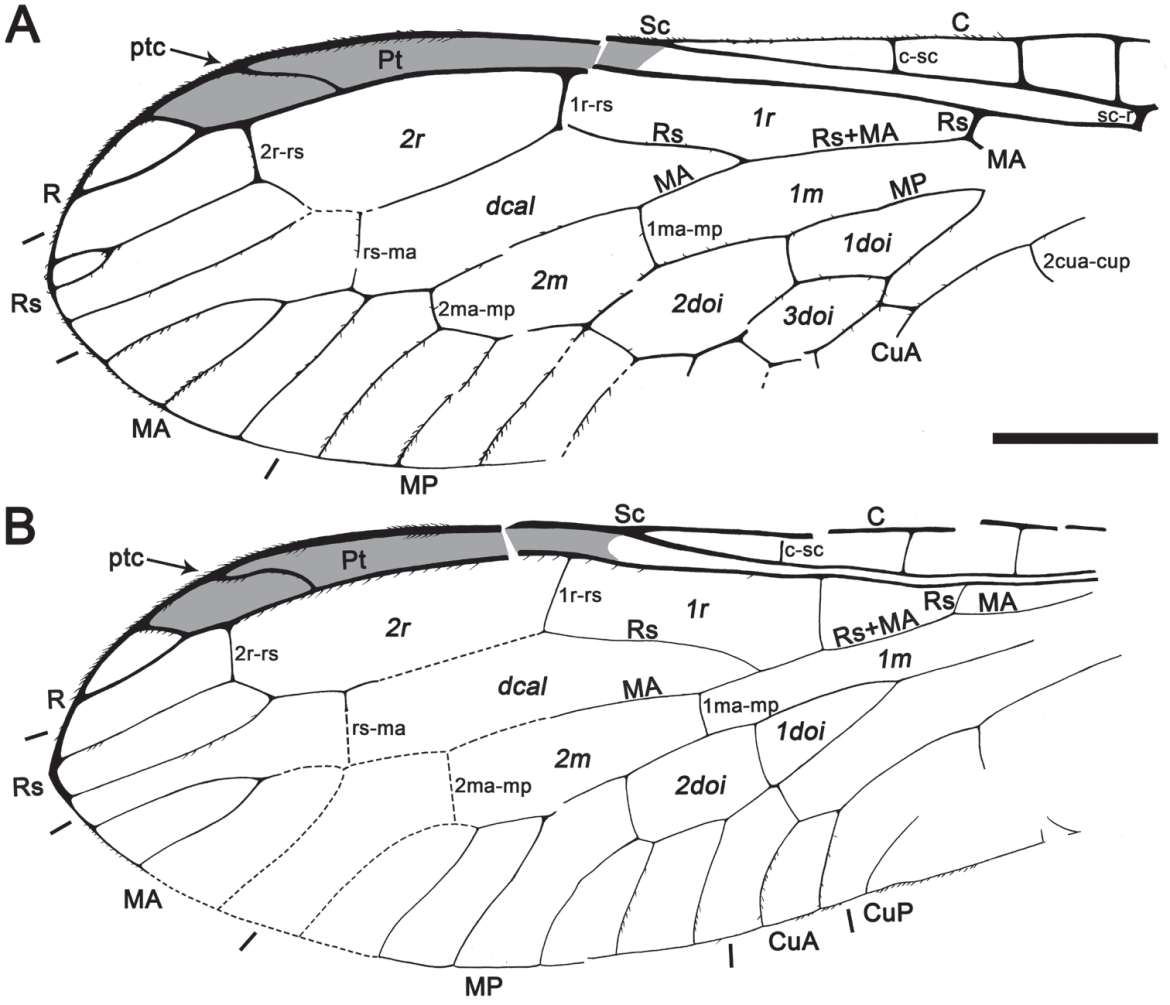
Drawings of *Necroraphidia arcuata* gen. et sp. n., holotype CES 391.1. **A** right forewing **B** right hind wing with reconstructed part taken from the left hind wing (dashed line). Only the distalmost c-sc crossvein has been tagged for both wings. Scale bar = 1 mm (both wings at the same scale).

##### Etymology.

The specific epithet is the Latin term *arcuatus*, meaning “bent”, and refers to the arcuate form of the pterostigmal crossvein.

### Genus *Styporaphidia* Engel & Ren, 2008

#### Styporaphidia ? hispanica

Pérez-de la Fuente, Peñalver, Delclòs & Engel sp. n.

urn:lsid:zoobank.org:act:0AAD19B5-EF08-4B10-B704-DBF6D56A0D6F

Fig. 6

##### Holotype.

MCNA 9343, from Peñacerrada I amber; hind wing fragment and abdominal apex, including the genitalia.

##### Diagnosis.

The new species is similar to *Styporaphidia magia* Engel and Ren, 2008 from the Late Jurassic of Inner Mongolia, China, in the presence of two pterostigmal crossveins. *Styporaphidia? hispanica* sp. n.differs in that the distance between 1ptc and 2ptc is three times the distance between 2ptc and the end of the pterostigma (two times in *Styporaphidia magia*), the forking of Rs at the apicalmost r-rs crossvein (rather than prior to it in *Styporaphidia magia*), and R meeting the apicalmost r-rs crossvein beyond the pterostigma (within in *Styporaphidia magia*).

##### Description.

**Figure 6.**
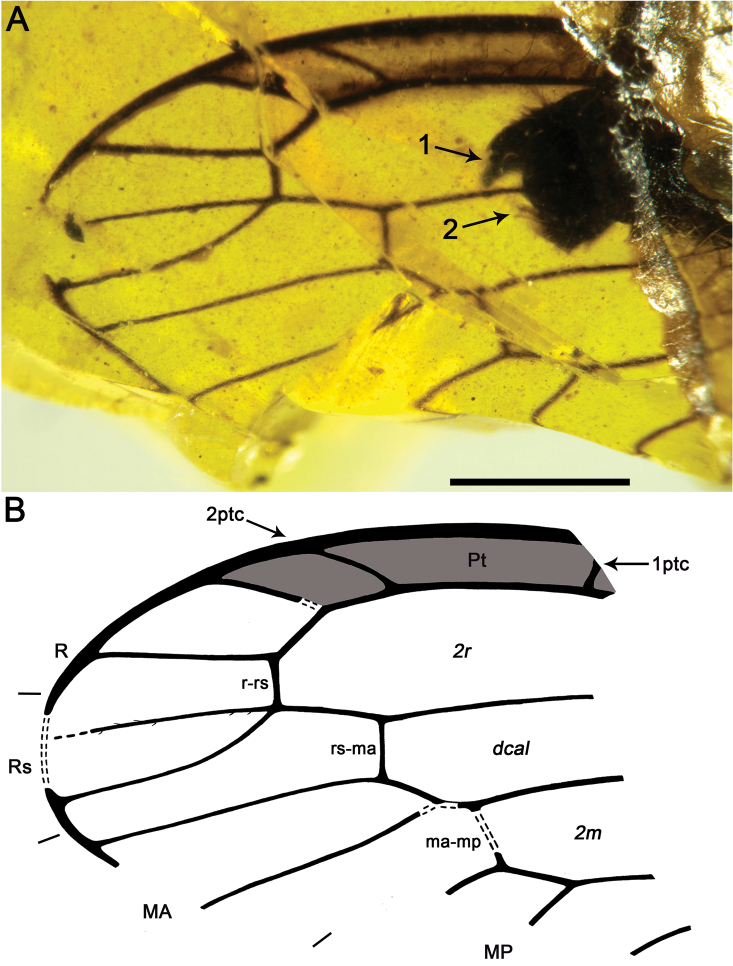
*Styporaphidia? hispanica* sp. n., holotype MCNA 9343. **A** hind wing apical fragment and genitalia; arrow 1 points to gonostyli 9, whereas arrow 2 points to the paired genital sclerites interpreted as the distalmost part of the parameres **B** drawing of preserved hind wing apical fragment. Scale bar = 1 mm.

Male. *Hind wing*. Length as preserved 3.5, maximum width as preserved 2.5; wing apex relatively rounded; C especially thick when compared to other veins; pterostigma almost with constant width along its entire length, infumate; pterostigma with two crossveins, distalmost crossvein oblique and slightly arcuate, proximal crossvein apparently straight, distance between 1ptc and 2ptc three times distance between 2ptc and end of pterostigma; Rs with two branches, forking at r-rs crossvein; R meeting apicalmost r-rs crossvein beyond pterostigma; rs-ma crossvein meeting MA after its distalmost fork; MA with two branches. *Abdomen*. Gonocoxites 9 with a few long setae;gonostyli 9 segment rather short, rounded (not acute), slightly upcurved;tergite 10 (+11?) with distal setae;paired, contiguous, acute genital structures located dorsad to gonostyli 9, interpreted as distalmost part of parameres (Fig. 6A).

##### Etymology.

The specific epithet refers to the occurrence of this species in ancient Spain (*Hispania* in Latin).

##### Comments.

Although the base of the pterostigma is not preserved and it is accordingly impossible to ascertain if it was diffuse (i.e., lacking a crossvein), this species is tentatively placed in the genus *Styporaphidia* owing to the presence of two pterostigmal crossveins. The presence of two pterostigmal crossveins is a rare feature among the Raphidioptera and otherwise known in a few other raphidiopterans, i.e, namely the baissopterids *Baissoptera kolosnitsynae* and *Baissoptera pulchra* (Martynova 1961: p. 81, fig. 7; Martins-Neto and Nel 1992: p. 428, figs 2, 3), but the present fossil is clearly not a baissopterid. The fragment is considered as corresponding to a hind wing due to the more distal disposition of both crossveins as occurs in *Styporaphidia magia* (the distance between them being greater than the distance between 2ptc and the end of the pterostigma), and by the relative position of the abdomen. The veins C and R appear to be especially thickened close to the wing apex as occurs also in *Styporaphidia magia* (Engel and Ren 2008: fig. 2), though this effect could be enhanced by the darkening of the margins of the pterostigma. Although the species is represented by a wing fragment and the genitalia, it is distinctive enough that it should be easy to associate with more complete material that may become available in the future.

#### Amarantoraphidia

Pérez-de la Fuente, Peñalver, Delclòs & Engel gen. n.

urn:lsid:zoobank.org:act:EA2F19D0-E3C8-43A7-BA92-BFC73C2FA208

http://species-id.net/wiki/Amarantoraphidia

##### Type species.

*Amarantoraphidia ventolina* sp. n.

##### Diagnosis.

Minute size. Head ovoid, with the portion posterior to the compound eyes longer than the eye diameter and tapering caudad; three large ocelli present, situated between anterior half of compound eyes; antennae with a low number of flagellomeres (i.e., ≤ 26). Pronotum slightly longer than head, with a constant height along its entire length. Mesotibiae especially swollen; process at midlength of the metatibiae absent. Forewing with costal field moderately broad; pterostigma elongate, without crossveins; Sc terminating into C slightly distad wing midlength; six c-sc crossveins present; two discoidal cells posterior to MP; apicalmost branch of CuA simple; 1cua-cup crossvein located at the M-CuA separation.

##### Etymology.

The new genus-group name is a combination of the Greek term *amarantos*, meaning “that never fades, ageless”, and *Raphidia*, common generic stem for snakeflies. The name is feminine.

##### Comments.

*Amarantoraphidia* gen. n.is first compared with the other described minute mesoraphidiid genera, which have a forewing length around 6 or less. They include amber inclusions but also some compression fossils. Apart from the minute size, in all of these taxa the pterostigma is very elongate, without crossveins, and basally closed by a crossvein. Regarding those as amber inclusions, *Amarantoraphidia* is readily distinct from the other two currently described Spanish mesoraphidiid genera, *Cantabroraphidia* and *Alavaraphidia* gen. n., as well as *Lebanoraphidia*, by its ovoid head shape (subquadrate in *Cantabroraphidia*, and rhomboidal in the other two genera) and by its lesser number of antennal flagellomeres (26 flagellomeres in *Cantabroraphidia*, 38 flagellomeres or more in *Lebanoraphidia*, and 44 in *Alavaraphidia*). The two genera *Nanoraphidia* (type species *Nanoraphidia electroburmica* Engel, 2002, Burmese amber, latest Albian in age) and *Grimaldiraphidia* (type species *Grimaldiraphidia luzzi* (Grimaldi, 2000), New Jersey amber, Turonian in age)share with *Amarantoraphidia* the ovoid head shape. However, in both of these genera the ocelli are placed between the posterior half of the compound eyes, not between the anterior part as in *Amarantoraphidia*. Additionally, whereas *Amarantoraphidia* has two discoidal cells posterior to MP in the forewing, *Grimaldiraphidia* has three and *Nanoraphidia* just a single cell. Also, in *Nanoraphidia* the M-CuA separation is located near midpoint of the first and second cua-cup crossveins (Engel 2002; Jepson et al. 2011), not at the 1cua-cup crossvein as in *Amarantoraphidia*.

In addition to the aforementioned taxa, five mesoraphidiids with minute size have been hitherto described from compression fossils: *Grimaldiraphidia parvula* (Martynov, 1925), from Karatau (South Kazakhstan), Late Jurassic in age; *Grimaldiraphidia mitchelli* (Jepson, Coram and Jarzembowski, 2009), *Grimaldiraphidia purbeckensis* Jepson, Coram and Jarzembowski, 2009, and *Mesoraphidia websteri* Jepson, Coram and Jarzembowski, 2009, the three species from the Purbeck Limestone Group, Dorset (UK), Berriasian in age; and *Nanoraphidia lithographica* Jepson, Ansorge and Jarzembowski, 2011 (tentatively assigned to this genus), from El Montsec (Spain), Early Barremian in age. *Grimaldiraphidia parvula* (described from a complete specimen but its wings unresolved dorsoproximally) has more proximal positions of the fork of Rs and the 2r-rs and rs-ma crossveins than *Amarantoraphidia* (Martynov 1925: p. 242, figs 7–9). *Grimaldiraphidia mitchelli* (described from a wing, most likely a forewing, with not preserved base and pterostigma), although possessing two discoidal cells posterior to MP as in the forewing of *Amarantoraphidia*, has a venation somewhat different, with the second radial cell relatively wide compared to its length, and more proximal positions of the fork of Rs and the 2r-rs and rs-ma crossveins (Jepson et al. 2009). *Grimaldiraphidia purbeckensis* possesses three discoidal cells posterior to MP in the forewing, the M-CuA separation is located near midpoint of the first and second cua-cup crossveins, and has more proximal positions of the fork of Rs and the 2r-rs and rs-ma crossveins (ibid.). *Mesoraphidia websteri* (based on a hind wing) has the Sc ending beyond the first radial cell, relatively shorter second radial and discal cells, and a single discoidal cell (two discoidal cells posterior to MP in *Amarantoraphidia*) (ibid.). Lastly, *Nanoraphidia lithographica* shows only one discoidal cell posterior to MP in the forewing as in the type species for the genus, *Nanoraphidia electroburmica* (Jepson et al. 2011).

#### Amarantoraphidia ventolina

Pérez-de la Fuente, Peñalver, Delclòs & Engel sp. n.

urn:lsid:zoobank.org:act:79F0A7A3-789D-4B3F-8E34-A021073A86AD

http://species-id.net/wiki/Amarantoraphidia_ventolina

Figs 7 [Fig F8] –9

##### Holotype.

CES 364.1, from El Soplao amber; almost complete female, just lacking the distalmost portion of both forewings beyond the end of the pterostigma and the distal third of the right hind wing. The first left leg is disarticulated. The specimen is preserved together with the following syninclusions: one evaniid (a new *Cretevania* species; CES 364.2, Pérez-de la Fuente et al. 2012, in prep.) and three other indeterminate hymenopterans, four dipterans (one chimeromyiid among them), one thysanopteran, a few charcoalified plant fibers, and a few timber debris, as well as other indeterminate organic remains.

##### Diagnosis.

As for the genus (*vide supra*).

##### Description.

**Figure 7.**
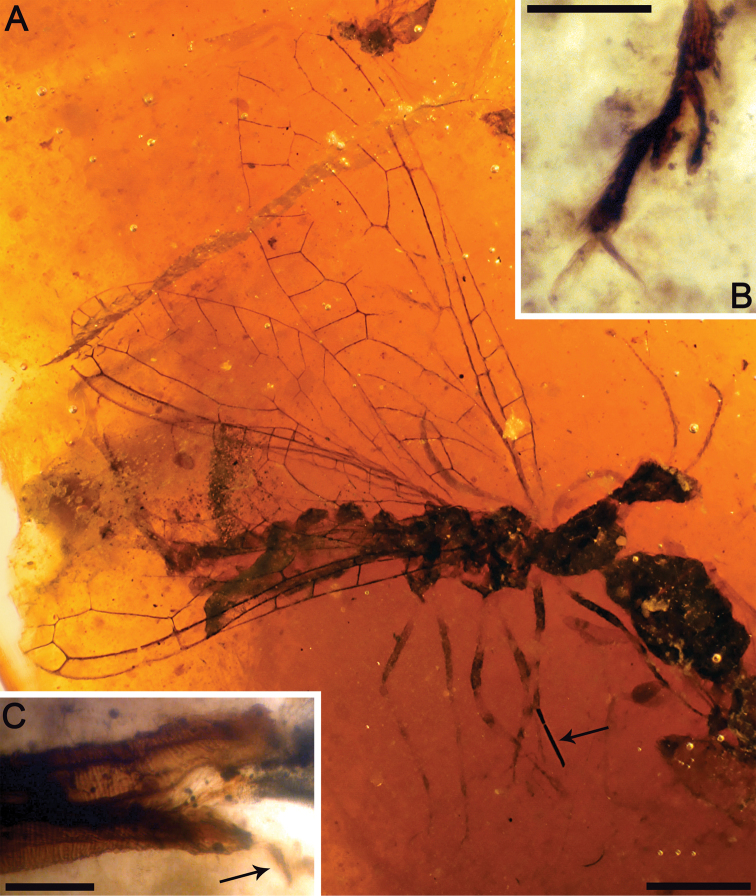
*Amarantoraphidia ventolina* gen. et sp. n., holotype CES 364.1. **A** lateral habitus, note a charcoalified plant fibernearby the specimen (arrow) **B** left metatarsi showing bilobed third tarsomere **C** distal portion of ovipositor, note its dense annulation; arrow points to a gonostylus. Scale bars: **A** = 1 mm; **B, C** = 0.1 mm.

Female.Integument dark brown; legs patterned as follows: femora darkened from just before their midlength to their end; three dark areas on tibiae, proximally, medially and distally; tarsomere 1 not darkened, distal tarsomeres darkened. *Head*.Ovoid, about 0.7–0.8 long, with portion posterior to compound eyes longer than eye diameter and tapering caudad; three large ocelli present, situated between anterior half of compound eyes; mandibles with teeth not visible;palps short;compound eyes large and exopthalmic, separated by distance slightly greater than compound eye length; antennae inserted around anterior tangent of compound eyes (exact insertion not visible); scape and pedicel gracile, both measuring about length of four flagellomeres and being subequal in thickness to them; 24 flagellomeres present, slightly longer than wide, with sparse, minute setae; coronal ecdysial cleavage line not evident; posterior border of head with a distinct collar-like lip.*Thorax*.Prothorax about 1.1 long, meso- plus metathorax 1.4 long; pronotum slightly longer than head, with a constant height along its entire length (i.e., without a distinct change of slope in lateral view); a few spines visible on prothorax, dorsoanterior mesothorax apparently with a few small spines; all tibiae with apical spines; mesotibiae especially swollen; metatibiae significantly more elongate and thinner than the other tibiae; process at midlength of metatibiae absent; five tarsomeres, third with bilobed extensions lacking digitiform processes (Fig. 7B); pretarsal claws simple, with a basal enlargement; arolium large.Wing veins brown, meeting wing margins without bifurcating; veins with strong, very short setae, especially abundant on C; membrane hyaline. *Forewing*.Length about 5.6 (tip not preserved), maximum width 1.9; costal field moderately broad (at widest point costal field about 1.4 wider than pterostigma); six c-sc crossveins present, two basalmost c-sc crossveins particularly close to each other; Sc terminating into C slightly distad wing midlength; single, proximal sc-r crossvein; pterostigma elongate (1.5 long), slightly longer than either radial cell; pterostigma with constant width along its entire length, faintly infumate, starting 0.4 (about three times pterostigmal width) beyond termination of Sc; pterostigma without crossveins, basally closed by a crossvein; Rs with two branches; two large radial cells present; first radial cell nearly as long as second radial cell, with MA arising slightly distad its midpoint; MA with two branches; two discoidal cells posterior to MP; apicalmost branch of CuA unforked; 1cua-cup crossvein located at M-CuA separation; 2A arcuate; jugal lobe not visible. *Hind wing*. Length about 4.1, maximum width 1.3; costal field distinctly narrower than in forewing; four c-sc crossveins present; sc-r crossvein absent; pterostigma elongate (1.5 long), slightly longer than second radial cell; pterostigma with about a constant width along its entire length, faintly infumate, starting 0.3 (slightly more than two times pterostigmal width) beyond termination of Sc; pterostigma without crossveins, basally closed by a crossvein; Rs with two branches; two radials cells present; MA with two branches; two discoidal cells posterior to MP, first one smaller and trianguloid; 1ma-mp crossvein close to fork between Rs and MA; anal area folded. *Abdomen*. Length 2.4; ovipositor robust, 1.7 long as preserved, 0.1 thick (about 15 times as long as wide); ovipositor showing dense annulations (Fig. 7C); ovipositor with faint, stiff, short sensory setae along its entire length; ovipositor gonostyli most likely club-shaped.

**Figure 8. F8:**
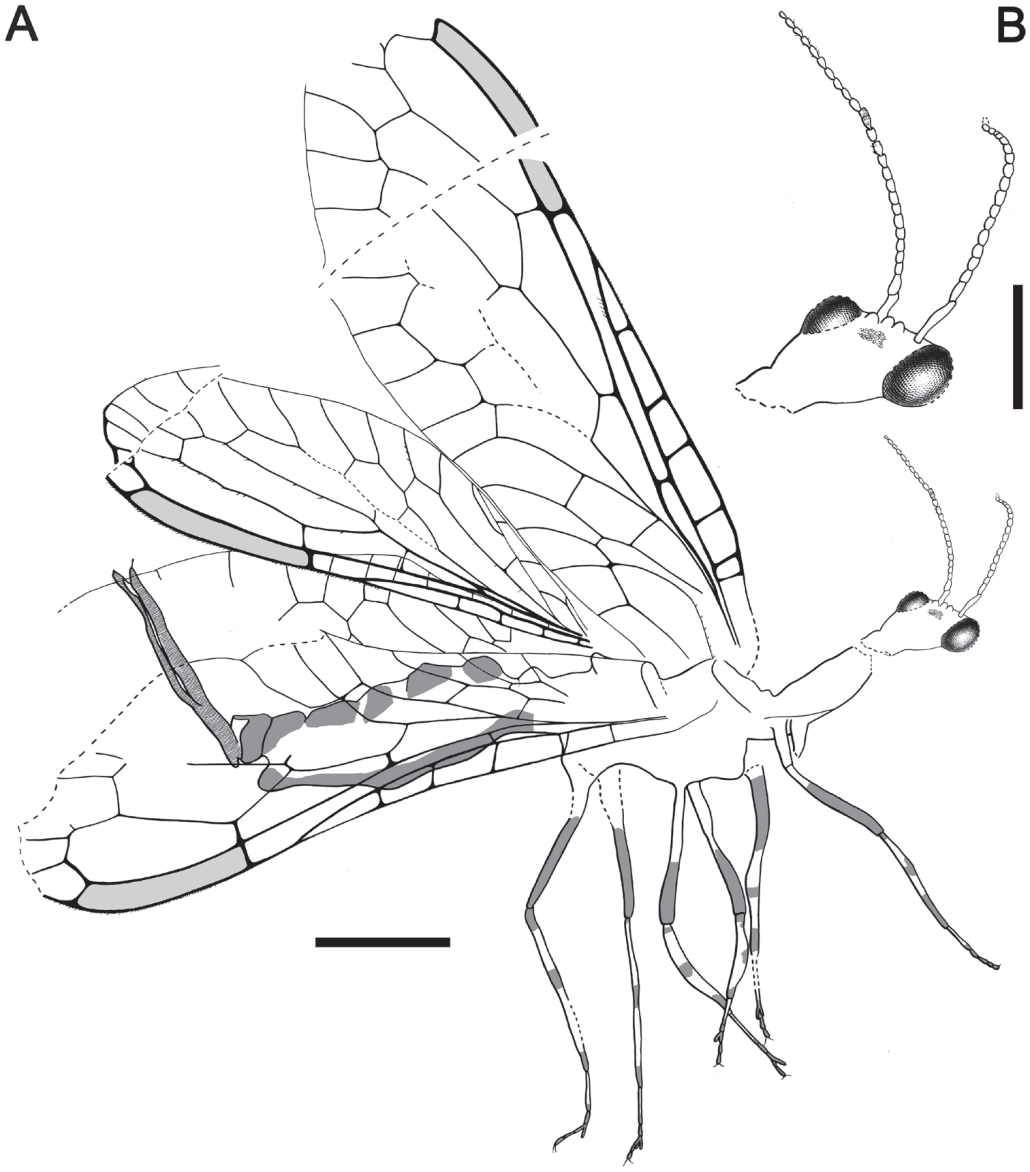
Drawings of *Amarantoraphidia ventolina* gen. et sp. n., holotype CES 364.1. **A** lateral habitus **B** head, magnified. Scale bars: **A** = 1 mm; **B** = 0.5 mm.

**Figure:**
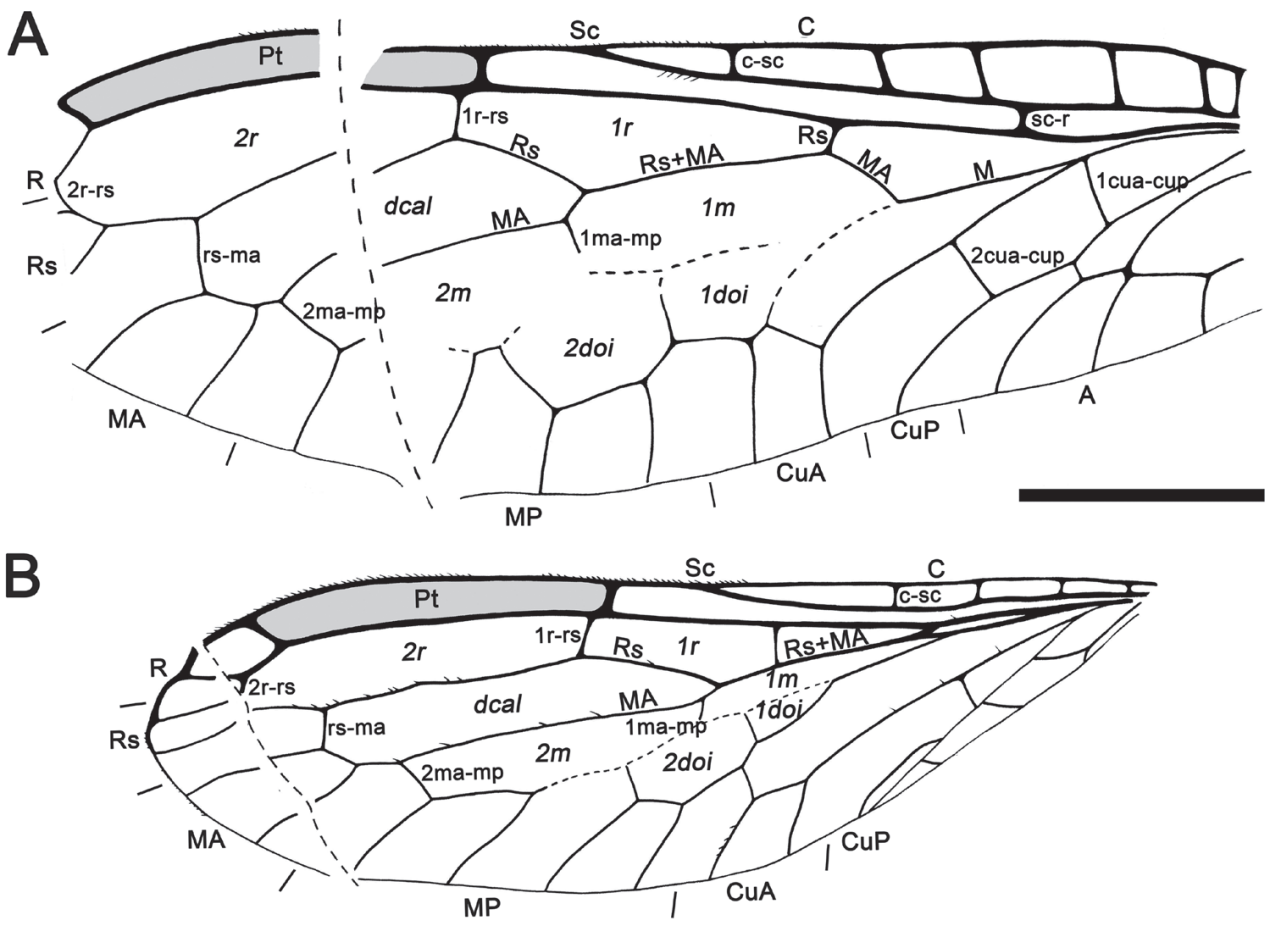
**Figure 9.** Drawings of *Amarantoraphidia ventolina* gen. et sp. n., holotype CES 364.1. **A** left forewing **B** left hind wing, depicted with its preservational folding, i.e., the basal part of MA is superimposed by the basal part of MP and the anal field is folded upwards. Only the distalmost c-sc crossvein has been tagged for both wings. Scale bar = 1 mm (both wings at the same scale).

##### Etymology.

In the Cantabrian mythology, the “ventolines” are tenacious and always cheerful fairy-like air beings that dwell in the depths of the sky and, when summoned, help defenseless fishermen by placidly steering their boats to the shore while embracing them with their warm green wings. The term has been singularized and feminized for combination.

##### Comments.

In extant snakeflies, the dense annulations of the ovipositor (cf. Fig. 7C) provide the flexibility necessary for introduction into irregular cavities, similar to a flexible metallic hose (Mickoleit 1973). It has been noted how mesoraphidiids would have had a shorter and thicker ovipositor than Recent Raphidioptera (Bechly and Wolf-Schwenninger 2011). The shape of the ovipositor in this specimen and also in *Alavaraphidia* gen. n. supports such a conclusion.

#### Alavaraphidia

Pérez-de la Fuente, Peñalver, Delclòs & Engel gen. n.

urn:lsid:zoobank.org:act:070A3E8E-1F78-4663-A909-1872E5EEA80E

http://species-id.net/wiki/Alavaraphidia

##### Type species.

*Alavaraphidia imperterrita* sp. n.

##### Diagnosis.

Minute size. Head rhomboidal, with clypeus especially elongate and the portion posterior to the compound eyes shorter than the eye diameter and strongly tapering caudad; three large ocelli present, situated near the posterior tangent of compound eyes; antennae extremely elongate, with a high number of flagellomeres (i.e., ≥ 38). Pronotum shorter than head, with a constant height along its entire length. All tibiae especially swollen medio-apically; process at midlength of the metatibia indistinct. Bilobed extensions of third tarsomeres with distal digitiform processes.

##### Etymology.

The new genus-group name is a combination of Álava, from Álava amber (the name of the group for Peñacerrada I and Peñacerrada II amber localities), and *Raphidia*, common generic stem for snakeflies. The name is feminine.

##### Comments.

**Figure 10.**
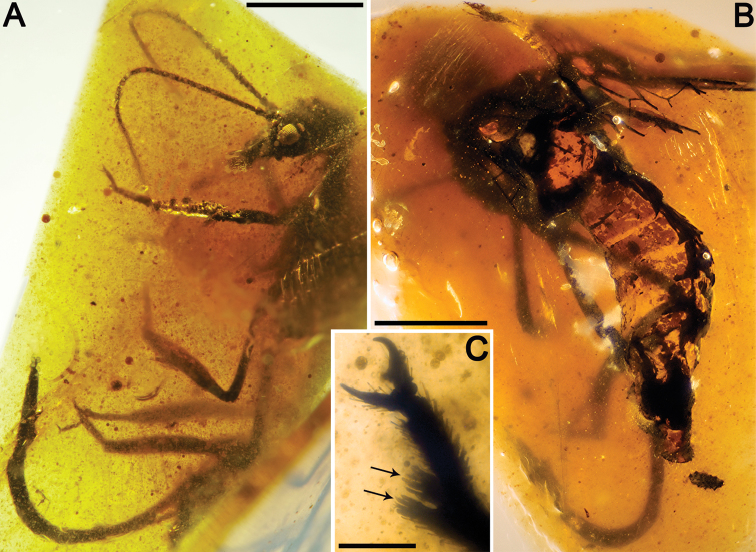
*Alavaraphidia imperterrita* gen. et sp. n., holotype MCNA 13608. **A** lateral habitus **B** dorsal habitus **C** left distal foretarsus, showing bilobed third tarsomere with digitiform processes (arrows). Scale bars: **A, B** = 1 mm; **C** = 0.1 mm.

Although most of the wings are not preserved, the other features of *Alavaraphidia* gen. n. are distinctive enough to justify the creation of a distinct taxon. The minute size of *Alavaraphidia* mainly limits its comparison to other minute taxa mainly described from amber inclusions (refer to comments for *Amarantoraphidia* gen. n.). Although a few other taxa based on wings from compressions are minute in size it is not possible to compare them with the new genus and species due to the absence of most of its wings. The genus *Lebanoraphidia* shares with *Alavaraphidia* the rhomboidal shape of the head with the compound eye length greater than that of the head posterior to the eyes (Bechly and Wolf-Schwenninger 2011); the head shape is ovoid in the genera *Grimaldiraphidia*, *Nanoraphidia*, and *Amarantoraphidia*,and subquadrangular in the genus *Cantabroraphidia*. Moreover, the genus *Lebanoraphidia* differs from *Alavaraphidia* in the lesser number of flagellomeres (note how the number of flagellomeres depicted for *Lebanoraphidia nana* Bechly and Wolf-Schwenninger, 2011, type and only species of the genus, seems to not match the description and photographs), the shorter clypeus, the shorter portion posterior to the compound eyes, the longer relative length of the pronotum, and the assumed absence of distal digitiform processes on the third tarsomere’s bilobed extensions. The high number of flagellomeres of *Alavaraphidia* (44) is most similar to that of *Lebanoraphidia*, itself with around 38 flagellomeres (Bechly and Wolf-Schwenninger 2011). Otherwise, 20 flagellomeres are present in the genus *Nanoraphidia* (Engel 2002), 23 in *Grimaldiraphidia* (Grimaldi 2000), 24 in *Amarantoraphidia*,and 26 in *Cantabroraphidia* (Pérez-de la Fuente et al. 2010). The distal digitiform processes from the third tarsomere’s bilobed extensions (fig. 10C) have not been reported from any other taxon and thus it is considered a unique character. Regarding the leg patterning, the medial dark patch found on the tibia in *Cantabroraphidia* (ibid.) and *Amarantoraphidia* is absent in *Alavaraphidia*. However, this character is not considered relevant at the generic level and hence has been discarded from the present diagnosis.

#### Alavaraphidia imperterrita

Pérez-de la Fuente, Peñalver, Delclòs & Engel sp. n.

urn:lsid:zoobank.org:act:E1406721-46BE-4F26-869E-EC6C5AE6CD01

http://species-id.net/wiki/Alavaraphidia_imperterrita

Figs 10 , 11

##### Holotype.

MCNA 13608, from Peñacerrada I amber; partial specimen showing head and ventral parts of thorax and abdomen, including the ovipositor. Only the basalmost part of the wings is preserved.

##### Diagnosis.

As for the genus (*vide supra*).

##### Description.

Female.Body length excluding ovipositor 5.7. Integument dark brown; legs patterned as follows: femora darkened from just before their midlength to their end; two dark regions on tibiae, proximally and distally (medial dark region absent); tarsomere 1 not darkened, distal tarsomeres darkened. *Head*. Rhomboidal, about 1.2 long, with portion posterior to compound eyes shorter than eye diameter (about 0.7 times) and strongly tapering caudad; three large ocelli present, situated near posterior tangent of compound eyes; mandible with teeth not visible; palps short; clypeus especially elongate; compound eyes separated by about compound eye length; three large ocelli present, situated near posterior tangent of compound eyes; antennae inserted posterior to clypealfrons sulcus, basad anterior tangent of compound eyes; antennae extremely elongate, with 44 flagellomeres; flagellomeres elongate, about 1.5 times longer than wide; scape and pedicel thicker than flagellomeres, scape measuring about two flagellomeres, pedicel measuring slightly more than a flagellomere; coronal ecdysial cleavage line not evident; posterior border of head not visible. *Thorax*. Prothorax about 0.8 long; meso- and metathorax about 1.1 long; pronotum shorter than head, with a constant height along its length (i.e., without a distinct change of slope in lateral view); thoracic dorsal spines not visible, if present; all tibiae especially swollen medio-apically, with apical spines, spines also visible on metatarsomeres; process at midlength of metatibia indistinct; five tarsomeres, third with bilobed extensions having six to eight distal digitiform processes (Fig. 10C), different in shape than regular leg setae (not tapering apically); pretarsal claws simple, with a basal enlargement; arolium large.Preserved wing veins brown, with strong, very short setae; preserved membrane hyaline.*Forewing*. Costal field not especially broad. Three c-sc crossveins preserved.*Hind wing*. Costal field distinctly narrower than in forewing. Three c-sc crossveins preserved.*Abdomen*.Length 2.7; ovipositor robust but rather elongate, about 2.7 long as preserved, 0.2 thick (about 15 times as long as wide);ovipositor with dense annulations; ovipositor with conspicuous faint, stiff, small sensory setae along its entire length; ovipositor gonostyli club-shaped; tergite 10 (+11?) with a distalmost stripe of stiff trichobothria (some probably from tergite 9).

**Figure 11.**
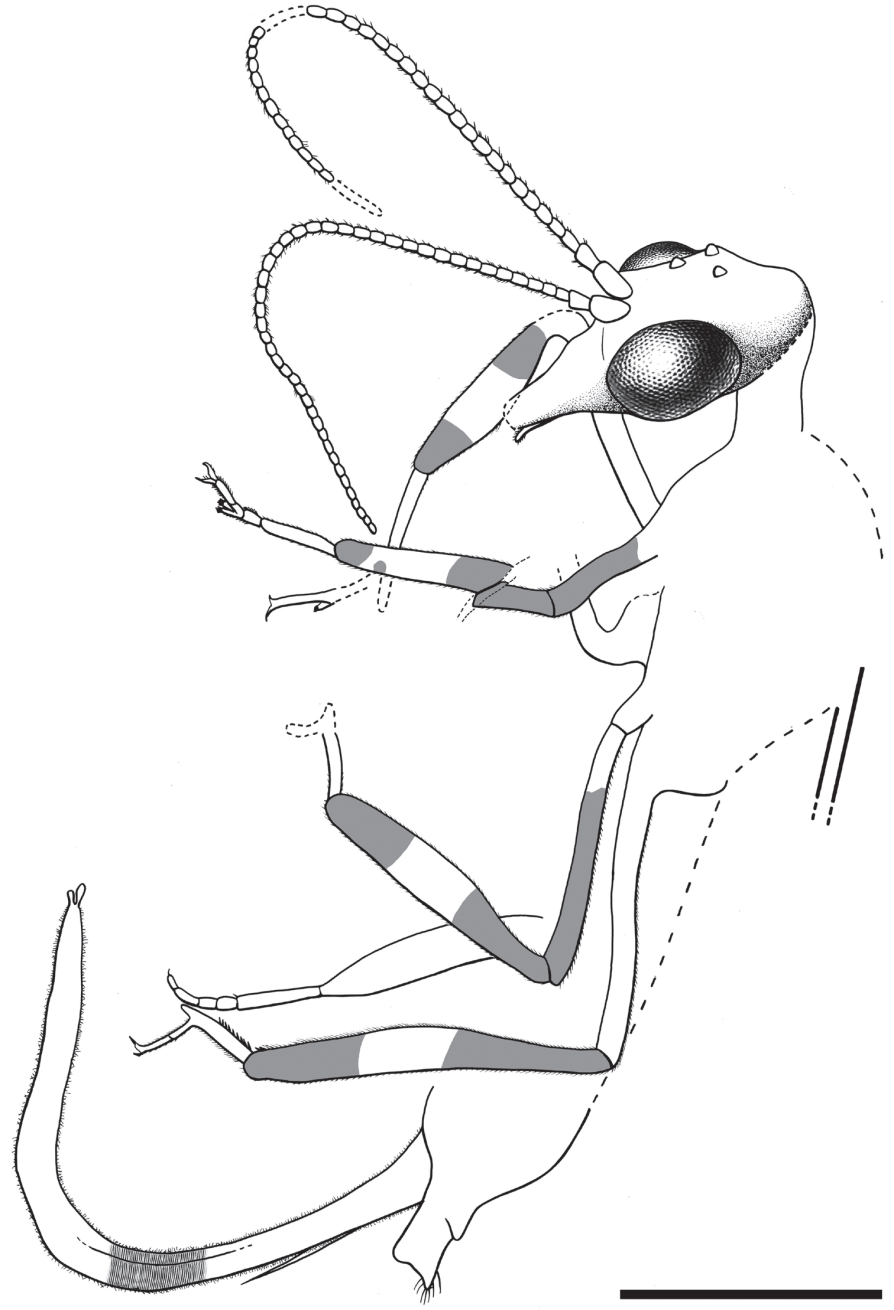
Drawing of *Alavaraphidia imperterrita* gen. et sp. n., holotype MCNA 13608. Lateral habitus. The annulations of the ovipositor have been depicted partially. Scale bar = 1 mm.

##### Etymology.

The specific epithet is the Latin term *imperterritus*, meaning “fearless”, and symbolizes the unalterable condition of an organism entrapped in amber.

#### Genus Cantabroraphidia

Pérez-de la Fuente, Nel, Peñalver & Delclòs, 2010

http://species-id.net/wiki/Cantabroraphidia

Fig. 12

Cantabroraphidia Pérez-de la Fuente, Nel, Peñalver, and Delclòs, 2010: 109. Type species: *Cantabroraphidia marcanoi* Pérez-de la Fuente, Nel, Peñalver, and Delclòs, 2010, by original designation.

##### Comments.

According to the original diagnosis, this monospecific genus was characterized by the following combination of characters: minute size (forewing length 5.5). Head more or less quadrangular; with portion posterior to the compound eyes slightly shorter than the eye diameter and not tapering caudad; three large ocelli present, situated between anterior half of the compound eyes; posterior border of head with a distinct collar-like lip. Pronotum subequal in length to head length, with anterior half narrowed dorsoventrally relative to posterior half (i.e., with slight downward curve in lateral view). Process present at midlength of the metatibiae in a posterior position. Forewing with costal field relatively broad (at widest point costal field as broad as pterostigma); pterostigma elongate, without crossveins; Sc terminating into C in distal two-thirds of wing length; four c-sc crossveins; two discoidal cells posterior to MP; 1cua-cup crossvein strongly basad M-CuA separation; posterior branch of MP forked. Refer to Pérez-de la Fuente et al. (2010) for a complete description of the type and only known species from El Soplao amber, based on an adult of unknown sex.

**Figure 12.**
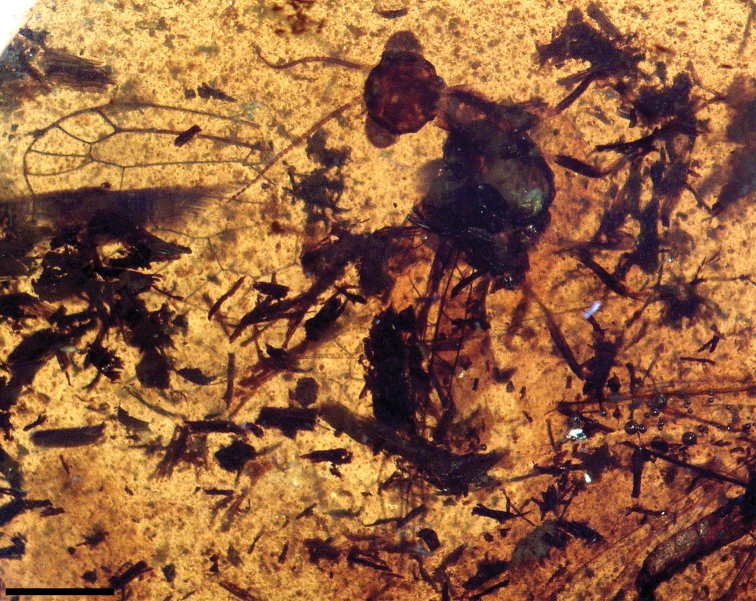
*Cantabroraphidia marcanoi* Pérez-de la Fuente et al., 2010. Laterodorsal habitus. Sex unknown. Note the abundant presence of timber debris in the amber piece, surrounding the specimen. Scale bar = 1 mm.

#### Genus and species indet. 1

Figs 13A , 14A

##### Material.

MCNA 9218, from Peñacerrada I amber; wing apex plus two minute wing fragments lacking formal descriptive significance. The sample consists of part and counterpart after the amber piece broke following the plane of the inclusion.

##### Descriptive notes.

Sex unknown. *Hind wing*(?). Length of preserved fragment 2.4,maximum width of preserved fragment 2.4;wing apex rounded; pterostigma slightly increasing in width distally, infumate; pterostigma with an almost straight subdistal crossvein; all apical branches relatively short; Rs with three branches, distalmost fork very short; apicalmost r-rs crossvein (2r-rs?) meeting R distal to the pterostigma; rs-ma crossvein situated at distalmost fork of MA; MA with two branches.

##### Comments.

The present material is distinct from other Spanish amber snakeflies but does not preserve enough detail to permit formal designation as a taxonomic entity. The presence of a pterostigmal crossvein immediately discounts *Cantabroraphidia* and *Amarantoraphidia* gen. n. MCNA 9218 highly resembles the hind wing of *Styporaphidia* in the shape and location of the distalmost pterostigmal crossvein. In fact, its venation is very similar to *Styporaphidia? hispanica* sp. n., though in the present material Rs is forked very close to the wing margin and the shape of its distalmost radial cell (most likely the second one) is somewhat different. Furthermore, the less pointed wing apex, shorter apical branches, and apical shapes of the radial, discal, and presumed second medial cells immediately distinguish MCNA 9218 from *Necroraphidia* gen. n. and genus and species indet. 2 (*vide infra*), and the almost straight pterostigmal crossvein and positions of the apicalmost r-rs (2r-rs?), rs-ma, and apicalmost ma-mp (2ma-mp?) crossveins further differentiate MCNA 9218 from *Necroraphidia*.

**Figure 13.**
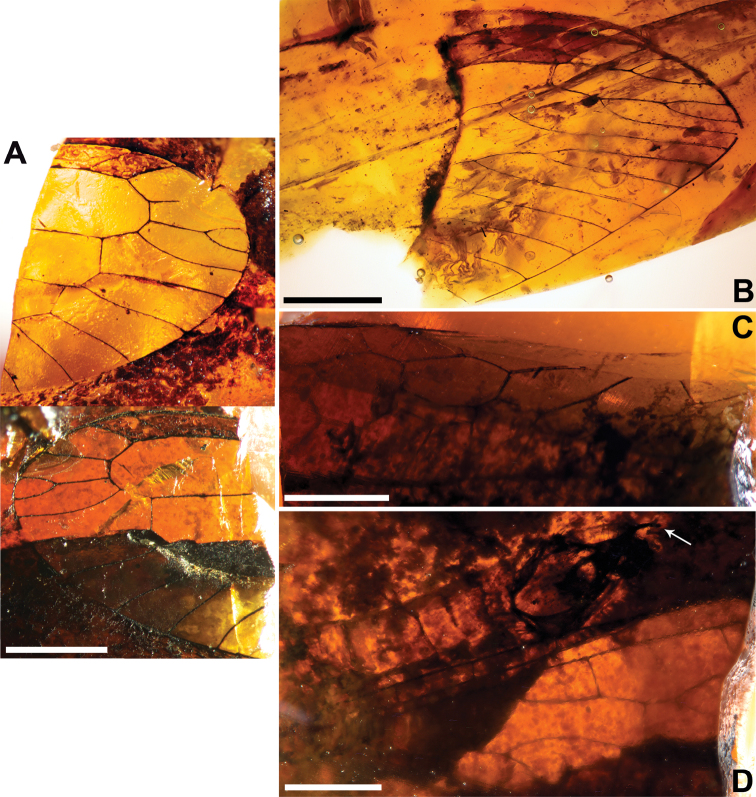
Fragmentary wing remains, genus and species indeterminate: CES 9218 part and counterpart (**A**), CES 376 (**B**), and MCNA 9316 (**C–D)**. **A** distal part of a hind wing(?), part (above) and counterpart (below) **B** preserved distal wing fragments **C** preserved part of forewing **D** preserved part of hind wing and distal part of abdomen. Arrow points to the tip of right gonostylus 9. Scale bars = 1 mm.

**Figure 14.**
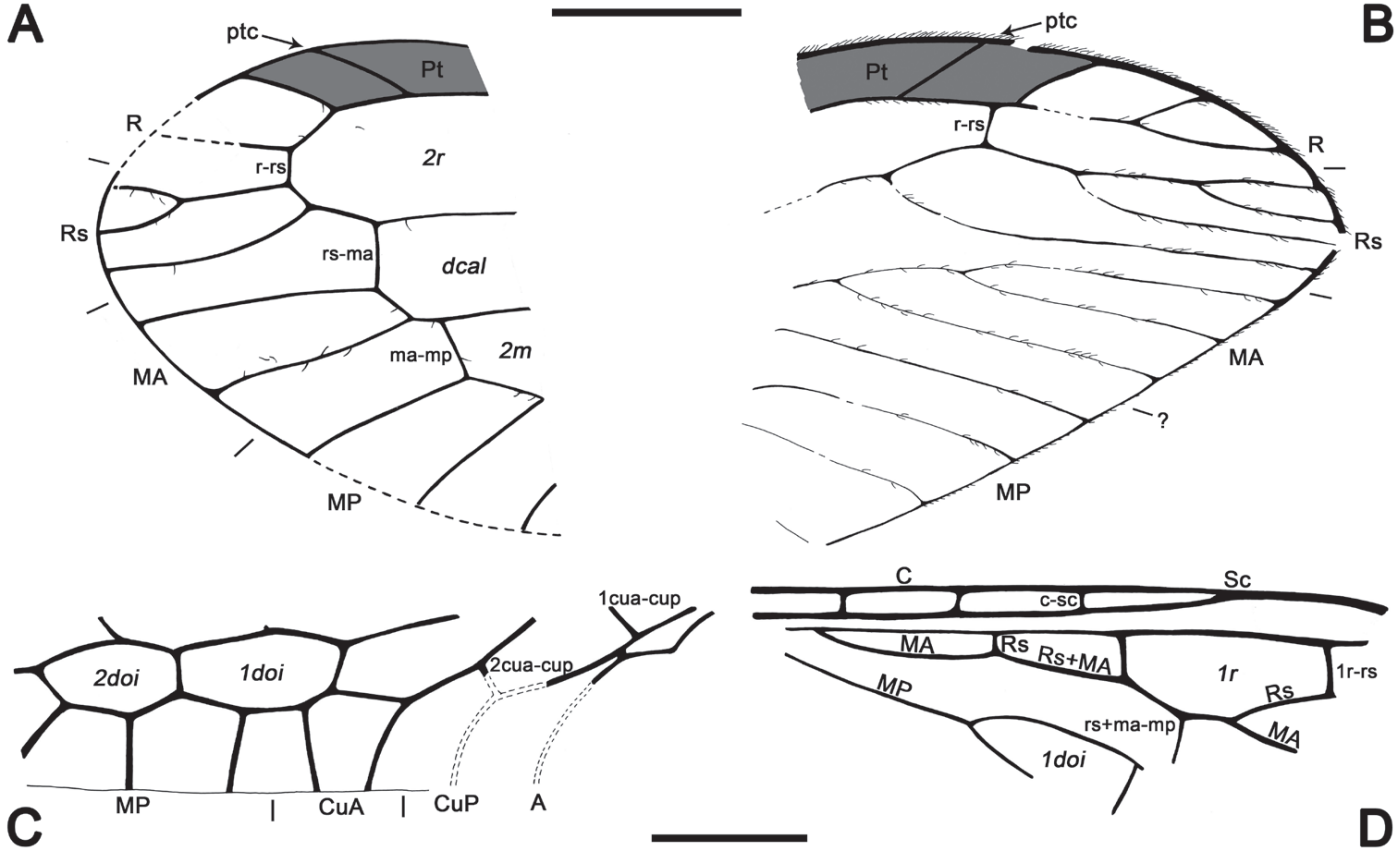
Drawings of fragmentary wing remains, genus and species indeterminate: CES 9218 counterpart (A), CES 376 (B), and MCNA 9316 (C–D). **A** distal part of hind wing(?) **B** best preserved distal wing fragment **C** preserved part of forewing **D** preserved part of hind wing; only the distalmost c-sc crossvein has been tagged; basalmost preserved c-sc crossvein not depicted. Scale bars = 1 mm (**A** and **B**; **C** and **D** at the same scale).

#### Genus and species indet. 2

Figs 13B , 14B

##### Material.

CES 376, from El Soplao amber; forewing apex and the area surrounding pterostigma from an additional wing. Some additional dorsoproximal parts of the wing are also present but with a very poor preservation, so just a few more characters can be elucidated. An indeterminate hymenopteran is present as a syninclusion.

##### Descriptive notes.

Length as preserved ca. 5.0 (wing well preserved only in 3.4 of that length), maximum width as preserved 2.7; wing apex pointed, positioned within Rs series; wing veins brown, meeting wing margins without bifurcating; veins with strong, short setae, especially abundant on C; Sc ending and proximal r-rs crossvein (1r-rs?; very faintly preserved) situated at about same wing length, pterostigma slightly widening distally, infumate; pterostigma with a very faint subdistal, rather straight (not conspicuously arcuate), strongly oblique crossvein; uncertain if pterostigmal division present; apical branches of Rs, MA and MP subparallel; two apical branches of R distal to pterostigma; Rs with four branches, posteriormost originating before distalmost r-rs crossvein (2r-rs?), separated from it by much more than its length; rs-ma and distalmost ma-mp crossveins lacking in preserved wing fragment, so most likely with a proximal position; MA most likely with three branches.

##### Comments.

Despite the fact that CES 376 is distinct from the other taxa in Spanish amber, it is not named as its preserved parts are not enough to resolve its affinities. The wing fragments show a high resemblance with some mesoraphidiids such as *Mesoraphidia obliquivenatica* (Ren, 1994) and *Caloraphidia glossophylla*, both from the Cretaceous compression deposit of Liaoning (China), as long as all of them share the presence of a strongly oblique, rather straight pterostigmal crossvein in a rather distal position and a distal portion of the wing with long apical branches and without crossveins other than the 2r-rs crossvein (Ren 1994: p. 134, fig. 4; 1997: p. 184, fig. 11). *Caloraphidia glossophylla* possesses subparallel apical branches of Rs, MA and MP, the Sc ending and the 1r-rs crossvein situated at about the same wing length, the 2r-rs crossvein closer to the end of the pterostigma than to the pterostigmal crossvein, and a pointed apex positioned within the Rs series, but in this species Rs has only three branches. By contrast, although *Mesoraphidia obliquivenatica* has Rs with four branches as in CES 376, the apical branches of Rs, MA and MP are not subparallel, the Sc ends in a more basal position than the 1r-rs crossvein, the 2r-rs crossvein is closer to the pterostigmal crossvein than to the end of the pterostigma, and the apex is more rounded and positioned between R and Rs. CES 376 is also quite similar to the wing apex of the compression fossil *Iberoraphidia dividua* Jepson, Ansorge and Jarzembowski, 2011, from El Montsec (Spain), Early Barremian in age, with its relatively simple venation, a Sc ending and a 1r-rs crossvein situated at about the same wing length, the 2r-rs, rs-ma and 2ma-mp crossveins not apically placed, the four branches of Rs, with the posteriormost branch of Rs originating before the 2r-rs crossvein, and the relatively simple apical fork of MA and MP (Jepson et al. 2011). CES 376 differs, however, in the presence of a pterostigmal crossvein (although in *Iberoraphidia dividua* the distal portion of the divided pterostigma could be basally closed by a crossvein), only two apical branches to R distal to the pterostigma (not three as in *Iberoraphidia dividua*), the posteriormost branch of Rs more proximally placed (separated from the distalmost r-rs by much more than its length, versus much shorter in *Iberoraphidia dividua*), and the more pointed wing apex which is positioned within the Rs series (rather than between R and Rs in *Iberoraphidia dividua*).

#### Genus and species indet. 3

Figs 13C–D , 14C–D

##### Material.

MCNA 9316, from Peñacerrada I amber; fore- and hind wing fragments from the same side of the body and a poorly preserved part of the abdomen, although the genitalia is somewhat visible. The amber piece contains abundant organic remains. The specimen is preserved together with legs of a spider as syninclusions.

##### Descriptive notes.

Male.Small size (inferred from preserved wing fragments). *Forewing*. Length as preserved about 4.5, maximum width not measurable. Two discoidal cells posterior to MP; posterior branch of MP unforked; M-CuA separation not preserved; 2A arcuate. *Hind wing*.Length as preservedabout 4.7, maximum width not measurable. Costal field relatively narrow; four c-sc crossveins preserved; Sc ending into C at length of first radial cell’s midlength; pterostigma not evidently infumate, not closed basally by a crossvein, at least proximally; first discoidal cell not especially small (or compact) (small in *Cantabroraphidia marcanoi*); rs+ma-mp crossvein present. *Abdomen*. Length as preserved (including genitalia) 3.8. Gonocoxites 9 with paired basal inner tubercles with small dark teeth; gonocoxites 9 elongate, with very long, stiff setae distally; gonostyli 9 very elongate, slightly upcurved; parameres not conspicuous; tergite 10 (+11?) with distalmost two or three stripes of trichobothria.

**Comments.** The preserved characters of MCNA 9316 are not enough to create a new taxon. The combined presence of two discoidal cells posterior to MP as in *Cantabroraphidia* and *Amarantoraphidia* gen. n., and the lack of a crossvein closing the pterostigma basally in the preserved wing length as in *Necroraphidia* gen. n., *Ororaphidia*, and *Styporaphidia* (showing three discoidal cells), precludes assignment to any of these genera. The lack of a crossvein closing the pterostigma basally can mean that the base of the pterostigma may have been diffuse or that the pterostigma may have been placed in a more distal position, as occurs in some mesoraphidiids. However, the inferred size of MCNA 9316 would have not been as reduced as in the minute mesoraphidiids but would have better fit with that of those mesoraphidiids with a diffuse pterostigmal base (refer to comments for *Necroraphidia* gen. n.). Furthermore, the presence of a rs+ma-mp crossvein in the hind wing as occurs in MCNA 9316 is a very rare character among described Mesoraphidiidae but more common in modern snakeflies, although it is also present in *Styporaphidia magia*. The morphology of the genitalia appear distinctive enough as to be recognizable in future findings. It is interesting to note that Aspöck and Aspöck (2004: figs 9, 10) used the presence of wartlike tubercles (covered with dark teeth) on the inner side of gonocoxite 9, larger but similar to those shown by MCNA 9316, as a diagnostic character for the inocelliid genus *Succinofibla* Aspöck & Aspöck, 2004 from Baltic amber. These authors also suggested that these tubercles could be related with the closing mechanism of the genitalia.

### Dichotomous keys

The following keys include only those species sufficiently diagnostic to name. As such, the additional morphospecies are not considered here and so all new material should be cross-referenced with the above accounts on those more fragmentarily known taxa. Note that *Alavaraphidia imperterrita* sp. n. is absent from the first key given that the diagnostic parts of the forewings are not preserved.

### Key to genera and species of Spanish amber Raphidioptera based on wings

**Table:** 

1	Fore-/hind wing with pterostigmal crossvein	2
–	Fore-/hind wing without pterostigmal crossvein (Mesoraphidiidae)	4
2	Fore-/hind wing with sparse or moderate crossvenation; at most with two radial cells, three medial cells, and three discoidal cells posterior to MP; often with no closed subradial cells (i.e., cells between Rs branches) (Mesoraphidiidae)	3
–	Fore-/hind wing with relatively rich crossvenation; with at least three radial cells, and numerous subradial, medial, and discoidal cells posterior to MP (Baissopteridae)	*Baissoptera? cretaceoelectra* sp. n.
3	Fore-/hind wing with a single pterostigmal crossvein; distalmost r-rs crossvein meeting R within pterostigma, Rs (proximally) forking before distalmost r-rs; rs-ma crossvein meeting MA before its distalmost fork	*Necroraphidia arcuata* gen. et sp. n.
–	Hind wing with two pterostigmal crossveins; distalmost r-rs crossvein meeting R beyond pterostigma, Rs forking at r-rs crossvein; rs-ma crossvein meeting MA after its distalmost fork	*Styporaphidia? hispanica* sp. n.
4	Forewing with four c-sc crossveins; apicalmost branch of CuA forked near wing margin; M-CuA separation between 1cua-cup and 2cua-cup crossveins (closer to latter)	*Cantabroraphidia marcanoi* Pérez-de la Fuente et al., 2010
–	Forewing with six c-sc crossveins; apicalmost branch of CuA unforked; M-CuA separation at the 1cua-cup crossvein	*Amarantoraphidia ventolina* gen. et sp. n.

### Key to minute mesoraphidiid amber genera based on non-wing characters

**Table:** 

1	Head not rhomboidal; compound eyes shorter, equal, or not much longer than head posterior to eyes; antennae with a low number of flagellomeres (≤ 26)	2
–	Head rhomboidal; compound eyes longer than head posterior to eyes; antennae very elongate, with a high number of flagellomeres (≥ 38)	5
2	Head ovoid; compound eyes shorter or equal than head posterior to eyes; antennae with less than 26 flagellomeres	3
–	Head quadrangular; compound eyes slightly longer than head posterior to eyes;antennae with 26 flagellomeres	*Cantabroraphidia* Pérez-de la Fuente et al., 2010
3	Compound eyes not distinctly shorter than head posterior to eyes; ocelli positioned between posterior half of compound eyes	4
–	Compound eyes distinctly shorter than head posterior to eyes; ocelli positioned between anterior half of compound eyes	*Amarantoraphidia* gen. n.
4	Compound eyes nearly equal than head posterior to eyes; antennae longer than head length; posterior border of head without a collar-like lip	*Nanoraphidia* Engel, 2002
–	Compound eyes apparently slightly longer or equal than head posterior to eyes; antennae shorter than head length; posterior border of head with a collar-like lip	*Grimaldiraphidia* Bechly & Wolf-Schwenninger, 2011
5	Compound eyes two times head posterior to eyes; clypeus short; antennae with around 38 flagellomeres; pronotum longer than head; bilobed extensions of third tarsomere lacking digitiform processes	*Lebanoraphidia* Bechly & Wolf-Schwenninger, 2011
–	Compound eyes ca. 1.4 times head posterior to eyes; clypeus especially elongate; antennae with more than 38 flagellomeres; pronotum shorter than head; bilobed extensions of third tarsomere with distal digitiform processes	*Alavaraphidia* gen. n.

## Discussion

### Taxonomy

Recently, Bechly and Wolf-Schwenninger (2011) transferred several species of *Mesoraphidia* to the genus *Grimaldiraphidia*. Based on their study, these transfers were made to eliminate the putative paraphyly of *Mesoraphidia*, and the referred species were claimed to share the synapomorphic characters the authors used for their tribe Nanoraphidiini Bechly and Wolf-Schwenninger, 2011 (although recognition of this tribe leaves the remainder of their Mesoraphidiinae paraphyletic), i.e, “Rs distally unbranched or only with single apical fork, [ptero]stigma very long, postorbital margin region of head shortened, ovipositor short and conspicuosly strong (?), minute size of body and wings”. While at least those few wing characters are correct for the combinations *Grimaldiraphidia mitchelli*, *Grimaldiraphidia parvula*, and *Grimaldiraphidia purbeckensis*, the transference of *Mesoraphidia durlstonensis* Jepson, Coram and Jarzembowski, 2009 and *Mesoraphidia heteroneura* Ren, 1997 to *Grimaldiraphidia* is in stark contradiction with some of these, as the holotype of *Mesoraphidia durlstonensis* has a forewing 10.6 long (Jepson et al. 2009), whereas *Mesoraphidia heteroneura* has a forewing with Rs forked twice and 10.5 long (Ren 1997). Moreover, the combination *Yanoraphidia gaoi* Ren in Ren et al., 1995 was restored in Pérez-de la Fuente et al. (2010) (it was previously transferred to *Mesoraphidia* by Engel in 2002) based on the distinctive pterostigma that goes up to the apex of R. By this reasoning, the transfer of this species by Bechly and Wolf-Schwenninger (2011) from the genus *Mesoraphidia* to the genus *Grimaldiraphidia* is not valid nor necessary. Accordingly, we formally return *Mesoraphidia gaoi* to *Yanoraphidia* (as done by Pérez-de la Fuente et al. 2010), and *Grimaldiraphidia durlstonensis* and *Grimaldiraphidia heteroneura* to *Mesoraphidia*, restoring the combinations *Yanoraphidia gaoi* Ren in Ren et al., 1995 stat. rest., *Mesoraphidia durlstonensis* Jepson, Coram and Jarzembowski, 2009 stat. rest., and *Mesoraphidia heteroneura* Ren, 1997 stat. rest. While we acknowledge that these last two species should perhaps be placed in a new genus on their own, if the criteria of Bechly and Wolf-Schwenniger (2011) were followed, herein we prefer to take a conservative stance as the genus *Mesoraphidia* is in need of serious revision and further subdivision or changes should be done in the context of such a comprehensive study.

### Xylophilous mesoraphiidids

Seven larval mesoraphidiids have been described up to now from five Cretaceous amber localities around the world (Table 2). This circumstance contrasts with the lack of larval records as compressions, mostly due to the decreased fidelity of preservation of the soft body tissues in such deposits. Taking into account that the larval record of the remainder of holometabolous orders is similarly not that abundant in Cretaceous ambers, the relatively high number of larval mesoraphidiids most likely indicates that at least some, if not all, were corticolous, i.e., they lived under bark, as is well known in extant snakeflies (Aspöck 2002). Therefore, one would expect that this ecological proximity to the resin sources, in addition to their active predatory behaviour, would have greatly increased the chances of larval mesoraphidiids to become embedded in resin. This inference is reinforced by the ovipositor structure in *Amarantoraphidia ventolina* sp. n. and *Alavaraphidia imperterrita* sp. n. Bothshow a dense annulation along the entire ovipositor (Fig. 7C), as occurs in Recent snakeflies (Mickoleit 1973), and which is an adaptation suitable for laying eggs deep inside irregular surfaces such as bark crevices. Today, whereas all known larval Inocellidae are corticolous, a significant number of Raphidiidae are terricolous as immatures, living in superficial layers of soil or detritus at the base of trees or shrubs (Aspöck 2002). The fact that the adult holotypes of *Cantabroraphidia marcanoi*, *Necroraphidia arcuata* sp. n., and *Amarantoraphidia ventolina* have associated timber debris as syninclusions (especially abundant in the amber piece containing *Cretoraphidia marcanoi*, Fig. 12), most likely indicates xylophilous activity.

### Uniqueness of the Spanish amber snakefly fauna

Like many moderate- to large-sized insects, snakeflies are difficult to find as amber inclusions, particularly complete specimens in Cretaceous ambers. It is not surprising that those individuals recovered as complete, or relatively complete, inclusions are among the smallest members of the order. Nonetheless, immatures and fragments of much larger snakefly species are also found as amber inclusions as evidenced by the rich Spanish fauna. What is more curious is that among the more abundant Cretaceous amber sources such as Lebanon, Myanmar, New Jersey, and Canada, the reported snakeflies remain relative rarities (Table 2; Engel pers. obs.), and the fragmentary remains of numerous, sometimes larger, species that are observed in the Spanish deposits are not seen in these other outcrops. Why should the Iberian fauna be so particularly rich? It is similarly enigmatic that as of yet the younger French ambers (late Albian to Cenomanian), with the exception of two larval head capsules from the late Albian Archingeay - Les Nouillers outcrop in southwestern France (Perrichot and Engel 2007), have not yielded significant raphidiopteran specimens despite the close geographic proximity of the sources. This dramatic faunal difference is seen in other groups. For example, ants are relatively diverse in French amber, but entirely absent in Spanish amber, while stigmaphronid wasps are most diverse in Spanish amber but missing from the deposits of France (Ortega-Blanco et al. 2011). These faunal singularities might have been caused by the insularity that the Iberian Plate had from the Early Jurassic to the Late Cretaceous (see Blakey 2011). However, whether these contrasting faunas are just a result of localized environmental differences or the younger age of the French deposits is not entirely clear. The difference in age is not that great and given the similarities in some of the ant taxa between French and Burmese ambers, it is peculiar that there should not be greater similarity between the French and Spanish amber faunas. Furthermore, the amber of Archingeay - Les Nouillers, which is the richest French amberdeposit from the Cretaceous, seems to be sampling a more litter fauna (Perrichot 2004), so it is plausible that in that particular case these observed faunal differences, to a greater or lesser extent, could be taphonomic in origin.

However, another hypothesis could explain the high abundance/diversity of snakeflies in Spanish amber, apart from its insularity. Perhaps this abundance is partly owing to the common occurrence of wildfires in the Spanish amber environment, inferred from evidence at several Spanish amber localities (see Najarro et al. 2010). The evidence of abundant charcoal associated with the Spanish ambers, the abundant amber masses with charcoalified plant fibers as inclusions (e.g., Fig. 4A), and the presence of gleicheniacean ferns remains, the last of which are primary succession pioneers following wildfires. After wildfires, there is abundant timber available for xylophagous or xylophilous insects, and resin production among surviving trees is greatly increased (e.g., Grimaldi 2000, Moretti et al. 2006, McKellar et al. 2011, Santolamazza-Carbone et al. 2011). As has been pointed out above, it seems most likely that mesoraphidiids had a xylophilous biology, adults laying their eggs under or in bark crevices (including dead timber), and both immatures and adults assuredly predators of xylophagous or xylophilous arthropods (analogous to living species which predate tree or shrub inhabiting insects). Thus, the combination of a xylophilous biology of adult and immature snakeflies and periodic wildfires might together result in greater resources for these species and at the same time a higher probability of them becoming ensnared in the increased resin flows. *Necroraphidia arcuata* and *Amarantoraphidia ventolina* are actually preserved with charcoalified plant fibers as syninclusions (see Figs 4A, 7A; Pérez-de la Fuente et al. 2012), highlighting that they were entrapped after an episode of burning. Nevertheless, wildfires seem unable to explain entirely the richness of the Spanish snakefly paleofauna, as these would have also been involved in the origin of the Turonian amber accumulation from New Jersey (Grimaldi et al. 2000).

## Conclusions

The discovery of the first amber baissopterid is especially remarkable. Furthermore, the presence of the family in the Iberian territory during the Early Cretaceous completes the remarkable preexisting paleogeographic baissopterid hiatus between the Cretaceous localities from eastern Asia and Brazil.

With the fossils herein described, the Albian amber of Spain currently harbors the greatest abundance and diversity of snakeflies in Cretaceous resins. As is also suggested by other paleoentolomological records, this significant snakefly paleodiversity may reflect a faunistic singularity of the Iberian territory during the Albian perhaps as a consequence of its geographic isolation during the Jurassic and the Cretaceous, or a combination of this with an environment resulting from episodic wildfires. Wildfires would have increased the availability of dead wood as a substrate for the xylophagous or xylophilous insects to develop, which, in turn, could have aided the increase in the xylophilous, predatory snakefly populations.

## Supplementary Material

XML Treatment for Baissoptera ? cretaceoelectra

XML Treatment for Necroraphidia

XML Treatment for Necroraphidia arcuata

XML Treatment for Styporaphidia ? hispanica

XML Treatment for Amarantoraphidia

XML Treatment for Amarantoraphidia ventolina

XML Treatment for Alavaraphidia

XML Treatment for Alavaraphidia imperterrita

XML Treatment for Cantabroraphidia

XML Treatment for Genus and species indet. 1

XML Treatment for Genus and species indet. 2

XML Treatment for Genus and species indet. 3
